# Novel mechanisms underlying inhibition of inflammation-induced angiogenesis by dexamethasone and gentamicin via PI3K/AKT/NF-κB/VEGF pathways in acute radiation proctitis

**DOI:** 10.1038/s41598-022-17981-8

**Published:** 2022-08-18

**Authors:** Yousong Li, Qin Ding, Jinsheng Gao, Chunxia Li, Pengxiao Hou, Jie Xu, Kaiqi Cao, Min Hu, Lin Cheng, Xixing Wang, Xiaoling Yang

**Affiliations:** 1 Department of Traditional Chinese Medicine, Shanxi Bethune Hospital, Shanxi Academy of Medical Sciences, Tongji Shanxi Hospital, Third Hospital of Shanxi Medical University, Taiyuan, 030032 China; 2 Cancer Center, Shanxi Bethune Hospital, Shanxi Academy of Medical Sciences, Tongji Shanxi Hospital, Third Hospital of Shanxi Medical University, Taiyuan, 030032 China; 3 Department of Oncology, Shanxi Province Research Institute of Traditional Chinese Medicine, Taiyuan, 030012 China; 4 Ping An Healthcare and Technology Company Limited, Shanghai, 200032 China; 5 Department of Geriatrics, Shanxi Bethune Hospital, Shanxi Academy of Medical Sciences, Tongji Shanxi Hospital, Third Hospital of Shanxi Medical University, Taiyuan, 030032 China; 6 Department of Thoracic Oncology, Shanxi Bethune Hospital, Shanxi Academy of Medical Sciences, Tongji Shanxi Hospital, Third Hospital of Shanxi Medical University, Taiyuan, 030032 China

**Keywords:** Biochemistry, Cancer, Cell biology, Molecular biology, Gastroenterology, Medical research, Oncology

## Abstract

Acute radiation proctitis (ARP) is one of the most common complications of pelvic radiotherapy attributed to radiation exposure. The mechanisms of ARP are related to inflammation, angiogenesis, and so on. In this study we evaluated the effect of dexamethasone (DXM) combined with gentamicin (GM) enema on ARP mice, and explored its possible mechanisms by transcriptome sequencing, western blot and immunohistochemistry. C57BL/6 mice were randomly divided into 3 groups: healthy control group, ARP model group, and DXM + GM enema treatment group. ARP mice were established by using a single 6 MV X-ray dose of 27 Gy pelvic local irradiation. Transcriptome sequencing results showed that 979 genes were co-upregulated and 445 genes were co-downregulated in ARP mice compared to healthy mice. According to gene ontology (GO) and kyoto encyclopedia of genes and genomes (KEGG) pathway enrichment analysis, we firstly found that PI3K/AKT/NF-κB/VEGF pathways were mostly correlated with the inflammation-induced angiogenesis in ARP mice. PI3K/AKT pathway leads to the activation of NF-κB, which promotes the transcription of VEGF and Bcl-2. Interestingly, symptoms and pathological changes of ARP mice were ameliorated by DXM + GM enema treatment. DXM + GM enema inhibited inflammation by downregulating NF-κB and upregulating AQP3, as well as inhibited angiogenesis by downregulating VEGF and AQP1 in ARP mice. Moreover, DXM + GM enema induced apoptosis by increasing Bax and suppressing Bcl-2. The novel mechanisms may be related to the downregulation of PI3K/AKT/NF-κB/VEGF pathways.

## Introduction

Radiation proctitis is a very common complication of pelvic radiotherapy attributed to radiation exposure which typically afflicts quality of life, results in radiotherapy failure and unfavorable prognosis^1,2^. Radiation proctitis is traditionally divided into acute radiation proctitis (ARP) and chronic radiation proctitis (CRP), which are up to 50–75% and 5%, respectively^3^. The mechanism of radiation proctitis is not completely clarified but several signaling pathways have been suggested as part of the pathogenesis of this disease, including inflammatory response^4^, angiogenesis^5–7^, immune system^4^, cell adhesion^8^, and extracellular matrix remodeling^9^. It is characterized by abdominal pain, mucous bloody diarrhea, as well as anal spasm and tenesmus^3^, even intestinal obstruction or perforation.

Endoscopic therapies and hyperbaric oxygen^10^ have been used to treat radiation proctitis. Some medicines have been developed to treat radiation proctitis, such as sulphasalazine, balsalazide, mesalazine^11,12^, formalin^13^, glucocorticoids (dexamethasone^14^, betamethasone^12^ and hydrocortisone^15^), antibiotics (metronidazole and ciprofloxacin)^13^, vitamins (A, E and C^16^), aloe vera^17,18^, and MSCs^19^. However, these therapies of radiation proctitis remain a relatively unsatisfactory and need to be further researched. Our previous study has found that the commonly accepted treatment for patients with radiation proctitis in China is dexamethasone (DXM) combined with gentamicin (GM)^20^. But in some extent, the combination of DXM and GM results in decrease in immunity of ARP mice^5^.

Our previous study showed that DXM and GM combination enema could alleviate the symptoms of ARP mice^5^. Inflammation and angiogenesis are thought to play key roles in ARP. Frequent mucous diarrhea often emerges in the early stage of ARP, while bloody diarrhea predominates in the late stage of ARP. We proposed that inflammation-induced angiogenesis caused the development of ARP, and found that DXM combined with GM inhibited a major inflammatory signaling factor nuclear factor-kappa B (NF-κB) and a key angiogenesis regulator vascular endothelial growth factor (VEGF). However, the mechanism underlying inhibition of inflammation-induced angiogenesis by DXM combined with GM remains unclear. Thus the present study aims to evaluate the effect of DXM combined with GM on ARP mice, and to further elucidate its molecular mechanisms by using transcriptome sequencing, western blot, and immunohistochemistry.

## Results

### DXM + GM enema ameliorates symptoms and decreases general signs sore of ARP mice

**Figure 1:**
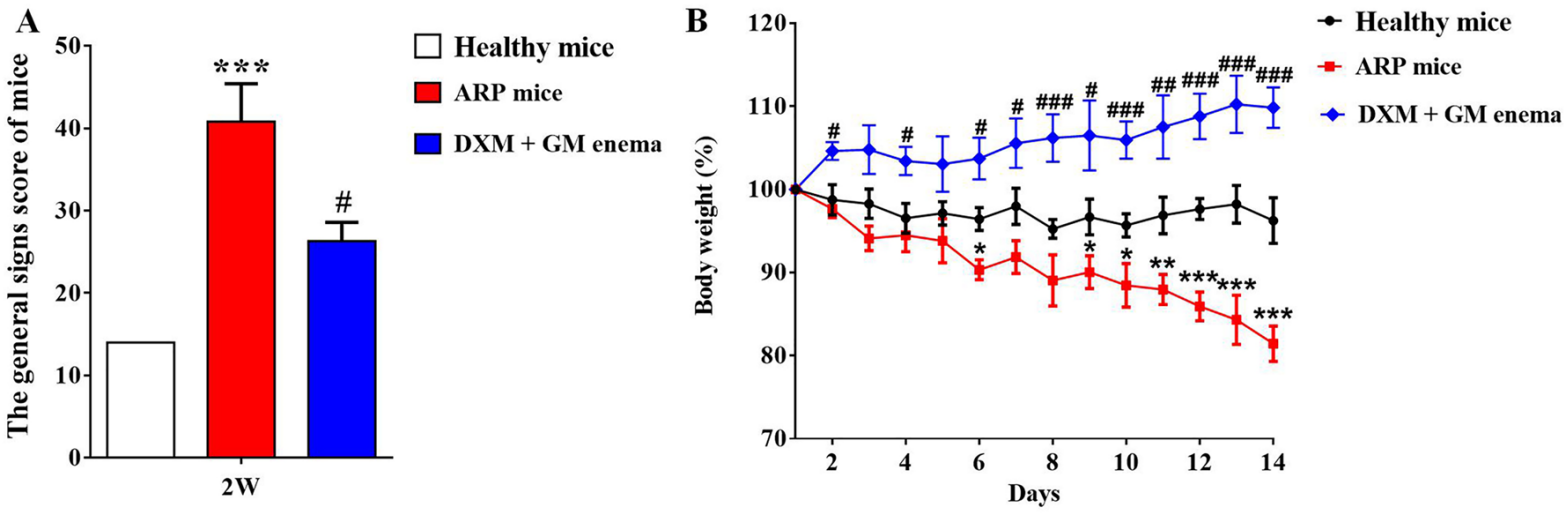
DXM + GM enema ameliorates symptoms and decreases general signs of ARP mice. (**A**) The difference in the general signs score of healthy mice and ARP mice at 2 weeks was significant (***P < 0.001). DXM + GM enema decreased the general signs score of ARP mice at 2 weeks (^#^*P* < 0.05). (**B**) The body weight of ARP mice was decreased compared with that of healthy mice at 6 days and 9–14 days (*P < 0.05, **P < 0.01, ***P < 0.001). Conversely, DXM + GM enema increased body weight of ARP mice at 2, 4 days and 6–14 days (^#^*P* < 0.05, ^##^*P* < 0.01, ^###^*P* < 0.001).

As shown in Fig. 1, there was no significant change in food consumption, water intake and body weight in healthy mice. Mice showed sustained reductions of food consumption, water intake and body weight in ARP model group. This result suggested that radiation not only damaged the rectal tissue of mice, but also affected the general signs of mice. DXM + GM enema ameliorated symptoms of ARP mice and decreased its general signs score (Fig. 1A). The body weight of ARP mice was decreased compared with that of healthy mice at 6 days and 9–14 days (Fig. 1B, *P < 0.05, **P < 0.01, ***P < 0.001). Conversely, DXM + GM enema increased body weight of ARP mice at 2, 4 days and 6–14 days (Fig. 1B, ^#^*P* < 0.05, ^##^*P* < 0.01, ^###^*P* < 0.001).

### DXM + GM enema alleviates pathological damage and decreases pathological grade of ARP mice

No obvious rectal tissue damage was observed in healthy control group mice. There were a large number of inflammatory cells infiltration, congestion and edema of rectal mucosa and submucosa, loss and necrosis of some glands, and structural disorders in rectal tissue of ARP mice (Fig. 7A top row). The pathological manifestations of ARP mice treated by DXM combined with GM were better than those of ARP mice (Fig. 7A top row). As Table 1 showed, the pathological grade of ARP mice was increased compared with healthy control group mice (***P < 0.001), indicating that ARP mice model was established successfully and the rectal tissue of mice was seriously damaged by radiation. DXM + GM enema alleviated pathological damage and reduced pathological grade of ARP mice (^###^*P* < 0.001).

**Table 1:** The pathological grade of rectal tissue of mice

Pathological grade	Healthy mice	ARP mice	DXM+GM eneman
Grade 0	10	0	0
Grade 1	0	0	4
Grade 2	0	1	2
Grade 3	0	3	3
Grade 4	0	6	1
Rank sum	11.078	6.935***	4.143^###^

Compared to healthy mice, ***P < 0.001, compared to ARP mice, ^###^*P *< 0.001, Rank-Sum test.

DXM + GM enema alleviates pathological damage and decreases pathological grade of ARP mice.

The rank sum test was used for the evaluation of pathological grade. The pathological grade of ARP mice was increased compared with healthy control group mice (***P < 0.001). DXM + GM enema alleviated pathological damage and reduced pathological grade of ARP mice (^###^*P* < 0.001).

### DXM + GM enema reduces spleen index while has no effect on liver index

**Figure 2:**
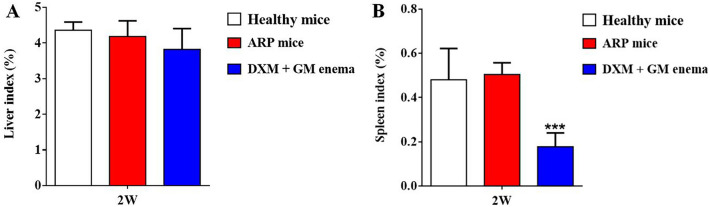
DXM + GM enema reduces spleen index while has no effect on liver index. (**A**) There was no significant difference in liver index at 2 weeks among healthy mice and ARP mice treated with or without DXM + GM enema. (**B**) Compared with healthy control group mice, the spleen index of mice treated by DXM + GM enema at 2 weeks was reduced in response to radiation (***P < 0.001).

As shown in Fig. 2A, there was no significant difference in liver index at 2 weeks among healthy mice and ARP mice treated with or without DXM + GM enema. Compared with healthy control group mice, the spleen index of mice treated by DXM + GM enema at 2 weeks was reduced in response to radiation (Fig. 2B, ***P < 0.001). Therefore, long-term use of the DXM + GM enema may affect the role of the spleen in immunity.

### DXM + GM enema regulates genes related to inflammation and angiogenesis in ARP mice

**Figure 3:**
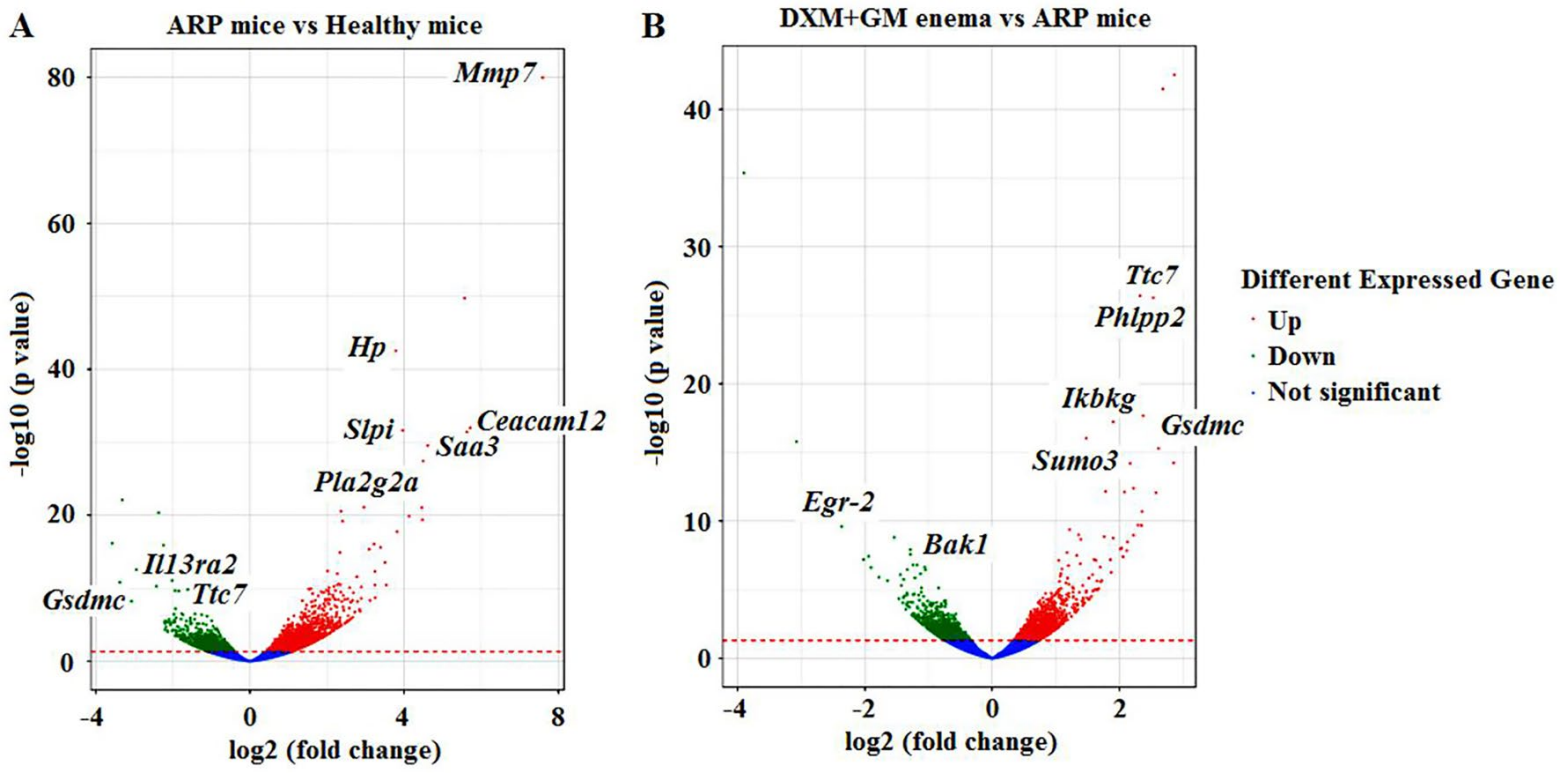
DXM + GM enema regulates the inflammation-induced angiogenesis in ARP mice. Transcriptome sequencing was used to analyze the difference in expression of signaling pathways between healthy mice and ARP mice treated with or without DXM + GM enema. (**A**) Volcano plots of differentially expressed genes between ARP mice and healthy mice. In the volcano plots, each dot is a gene, the “red” genes are those were significantly up-regulated while the “green” genes are those were significantly down-regulated in ARP mice compared with healthy mice at a q value < 0.05. However, the “blue” genes shown there were no significantly difference between ARP mice and healthy mice. In total, 1424 genes were identified as differentially expressed, including 979 genes those were up-regulated and 445 genes that were down-regulated. (**B**) Volcano plots of differentially expressed genes in ARP mice treated with or without DXM + GM enema. In total, 587 genes were identified as differentially expressed, including 348 genes those were up-regulated and 239 genes that were down-regulated.

Transcriptome sequencing was used to analyze the difference in expression of signaling pathways between healthy mice and ARP mice treated with or without DXM + GM enema. The profile of all different expressed genes is shown in Fig. 3. There were 1424 differential genes in ARP mice compared to healthy control group mice rectal tissues (Fig. 3A). The number of upregulated genes was 979, whereas that of downregulated genes was 445 (Fig. 3A). Based on transcriptome sequencing data of ARP mice, we found that 15 of the 979 upregulated genes and 15 of the 445 downregulated genes were associated with the development of ARP. Among these 15 upregulated genes, *Mmp7* (matrix metalloproteinase-7), *Hp* (haptoglobin), *Saa3* (serum Amyloid A3), *Slpi* (secretory leukocyte protease inhibitor), *Ceacam12* (carcinoembryonic antigen-related cell adhesion molecule 12) and *Pla2g2a* (phospholipase A2 group IIA) were significantly increased in ARP mice. While among these 15 downregulated genes, *Il13ra2* (Interleukin 13 Receptor alpha 2), *Ttc7* (tetratricopeptide repeat domain 7) and *Gsdmc* (gasdermin C) were significantly decreased in ARP mice. There were 587 differential genes in ARP mice before and after treatment of DXM + GM enema (Fig. 3B). The number of upregulated genes was 348, whereas that of downregulated genes was 239 (Fig. 3B). Based on transcriptome sequencing data, we found that 15 of the 348 upregulated genes and 15 of the 239 downregulated genes were associated with the effects of DXM + GM enema treatment. Among these 15 upregulated genes, *Ttc7*, *Phlpp2* (PH domain and leucine-rich repeat protein phosphatase 2), *Ikbkg* (inhibitor of kappaB kinase gamma, formerly known as NF-kB essential modulator, NEMO), *Gsdmc* and *Sumo3* (small ubiquitin-like modifier 3) were significantly upregulated after treatment with DXM + GM enema. While among these 15 downregulated genes, *Egr-2* (early growth response 2) and *Bak1* (BCL2-antagonist/killer 1) were significantly downregulated after treatment with DXM + GM enema. PHLPP2 loss enhances NEMO ubiquitination and subsequent IKKβ phosphorylation, resulting in increased NF-κB-dependent transcription of multiple target genes.

### DXM + GM enema regulates inflammatory response, immune response and angiogenesis in ARP mice

**Figure 4:**
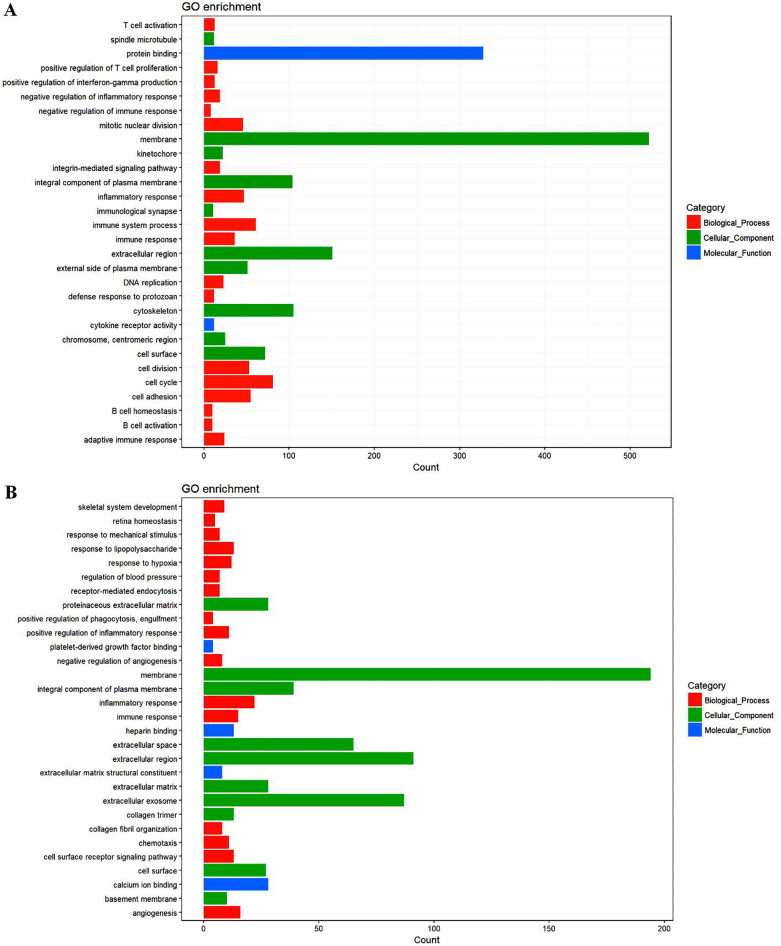
(**A**) Gene ontology classification of assembled unigenes in ARP mice compared with healthy mice. The DEGs genes were classified into three functional categories: biological process, cellular component and molecular function. The x-axis shows the number of genes in a category. The y-axis shows the specific category of genes in that main category. (**B**) Gene ontology classification of assembled unigenes in ARP mice treated with or without DXM + GM enema.

We conducted Gene ontology (GO) classification of assembled unigenes, and results showed that significant enrichment of genes involved in inflammatory response, immune system process, immune response and cell cycle was related to inflammation in ARP model group compared with healthy control group (Fig. 4A). And significant enrichment of genes involved in the inflammatory response, immune response and angiogenesis was related to inflammation-induced angiogenesis in DXM + GM enema treatment group compared with ARP model group (Fig. 4B).

### DXM + GM enema regulates PI3K/AKT signaling pathway and cytokine–cytokine receptor interaction in ARP mice

**Figure 5:**
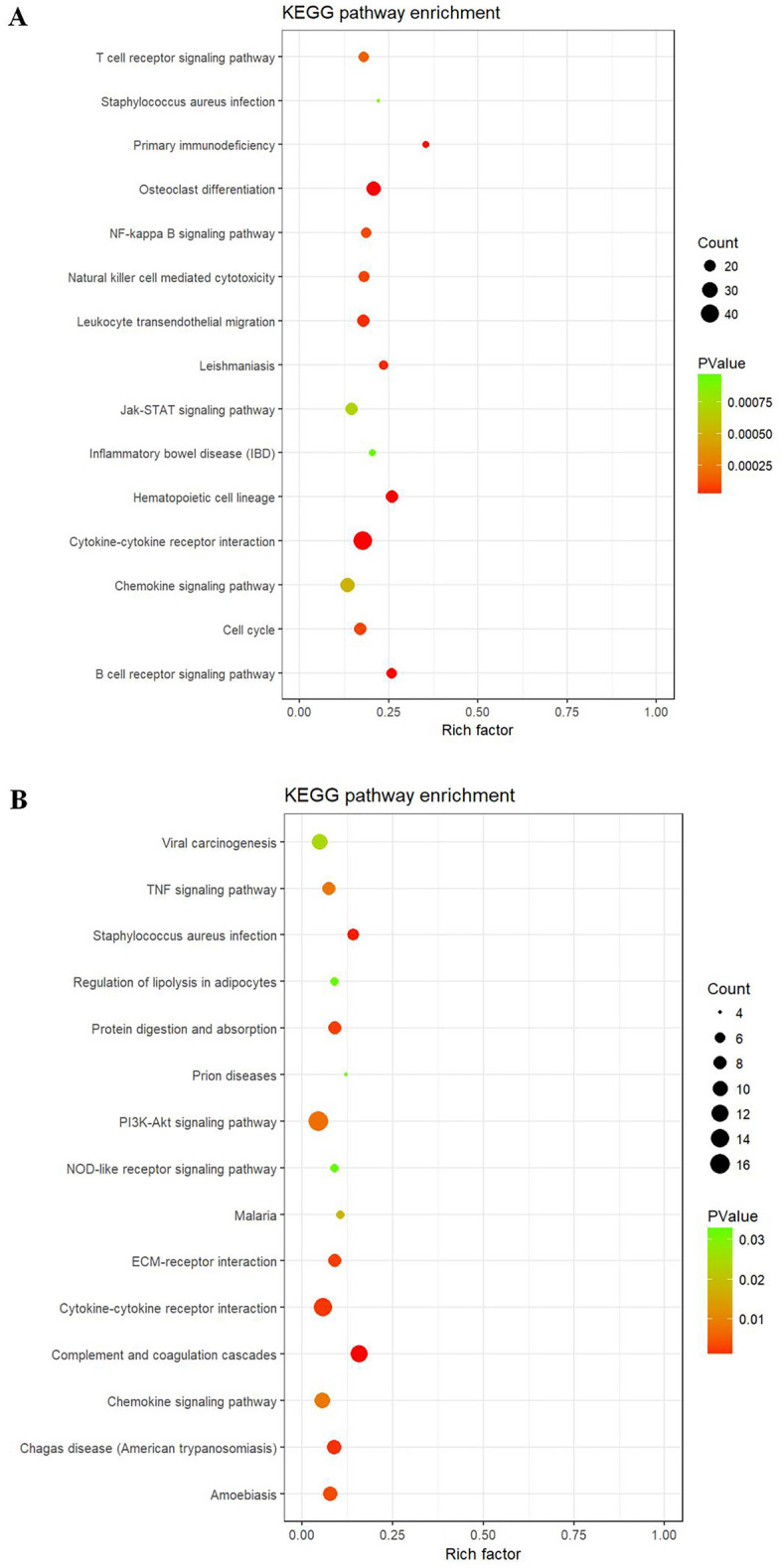
(**A**) KEGG pathway enrichment analysis showing genes involved in NF-κB signaling pathway and cytokine–cytokine receptor interaction were the largest in ARP mice compared with healthy mice. (**B**) KEGG pathway enrichment analysis showing genes involved in PI3K/AKT signaling pathway and cytokine–cytokine receptor interaction were the largest in DXM + GM enema treatment mice compared with ARP mice.

We further conducted KEGG analysis of differential genes, and results showed that the number of differential genes associated with NF-κB signaling pathway and cytokine–cytokine receptor interaction was the largest in ARP model group compared with healthy control group (Fig. 5A). And the number of differential genes associated with the PI3K/AKT signaling pathway and cytokine–cytokine receptor interaction was the largest in DXM + GM enema treatment group compared with ARP model group (Fig. 5B).

### DXM + GM enema ameliorates inflammation by inhibiting NF-κB signaling pathway in ARP mice

**Figure 6:**
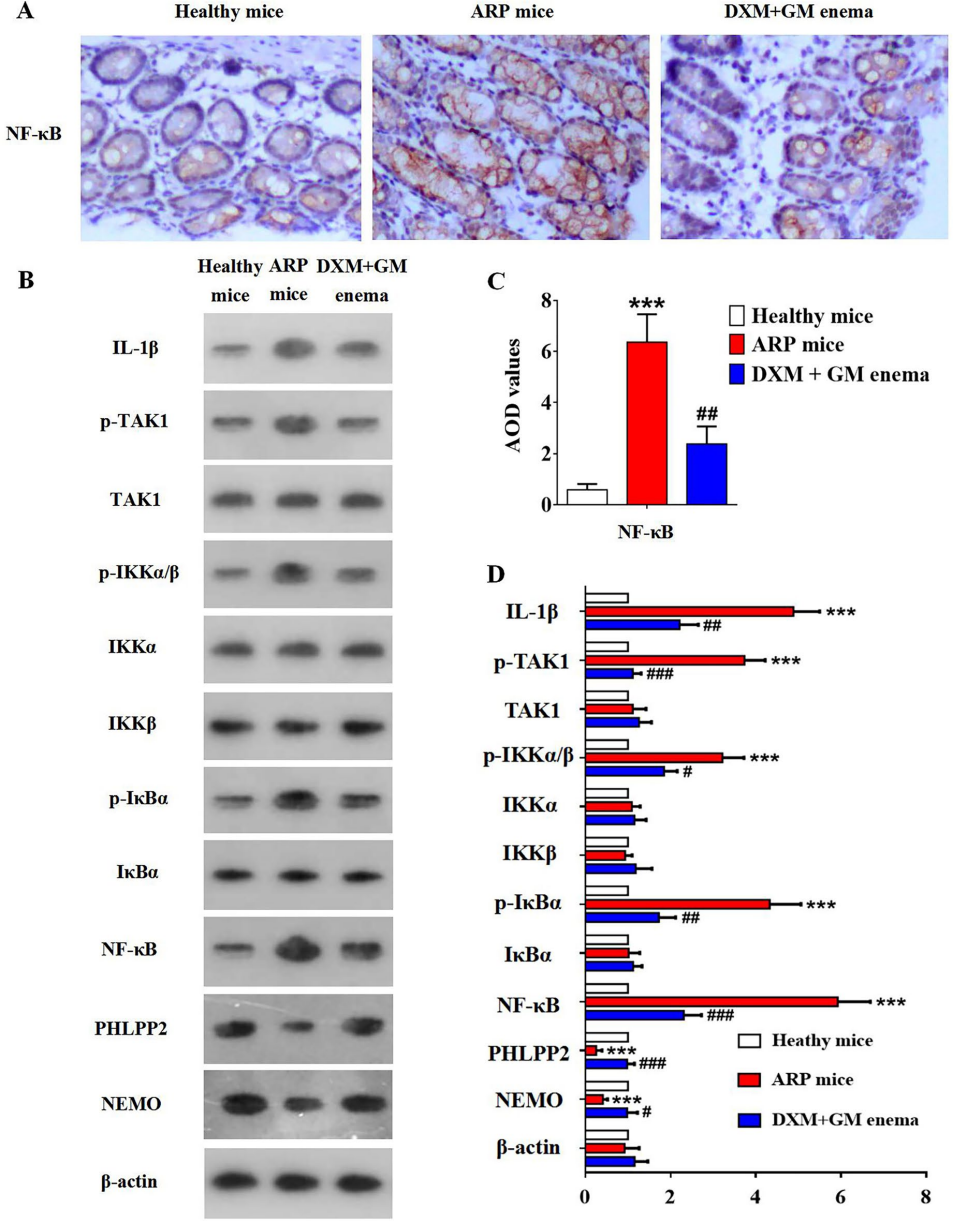
DXM + GM enema ameliorates inflammation by inhibiting NF-κB signaling pathway in ARP mice. (**A**) The immunohistochemistry of NF-κB in different groups of mice (n = 10 for each group) was observed under light microscopy (scale ×400). (**B**) The expression of NF-κB signaling pathway was tested by using western blot. (**C**) AOD values of NF-κB were detected by immunohistochemistry in different groups of mice (***P < 0.001, ^##^*P* < 0.01). (**D**) The expression of IL-1β, p-TAK1, p-IKKα/β, p-IκBα and NF-κB in rectal tissue of ARP mice was higher than that in healthy control group; however, the expression of PHLPP2 and NEMO was lower than that in healthy control group (***P < 0.001). DXM + GM enema inhibited the expression of IL-1β, p-TAK1, p-IKKα/β, p-IκBα and NF-κB, while up-regulated the expression of PHLPP2 and NEMO (^#^*P* < 0.05, ^##^*P* < 0.01, ^###^*P* < 0.001).

In order to assess whether DXM + GM enema possess anti-inflammation effects on ARP, NF-κB signaling pathway was examined in rectal tissue of ARP mice using western blot. Figure 6 showed that the expression of IL-1β, p-TAK1, p-IKKα/β, p-IκBα and NF-κB in rectal tissue of ARP mice was higher than that in healthy control group; however, the expression of PHLPP2 and NEMO was lower than that in healthy control group (Fig. 6B,D). These results suggested that NF-κB signaling pathway was activated in ARP mice (Fig. 6A,C). It was observed that DXM + GM enema inhibited the expression of IL-1β, p-TAK1, p-IKKα/β, p-IκBα and NF-κB, while up-regulated the expression of PHLPP2 and NEMO (Fig. 6B,D).

### DXM + GM enema inhibits angiogenesis by downregulating VEGF and AQP1 and inhibits inflammation by upregulating AQP3 in ARP mice

**Figure 7:**
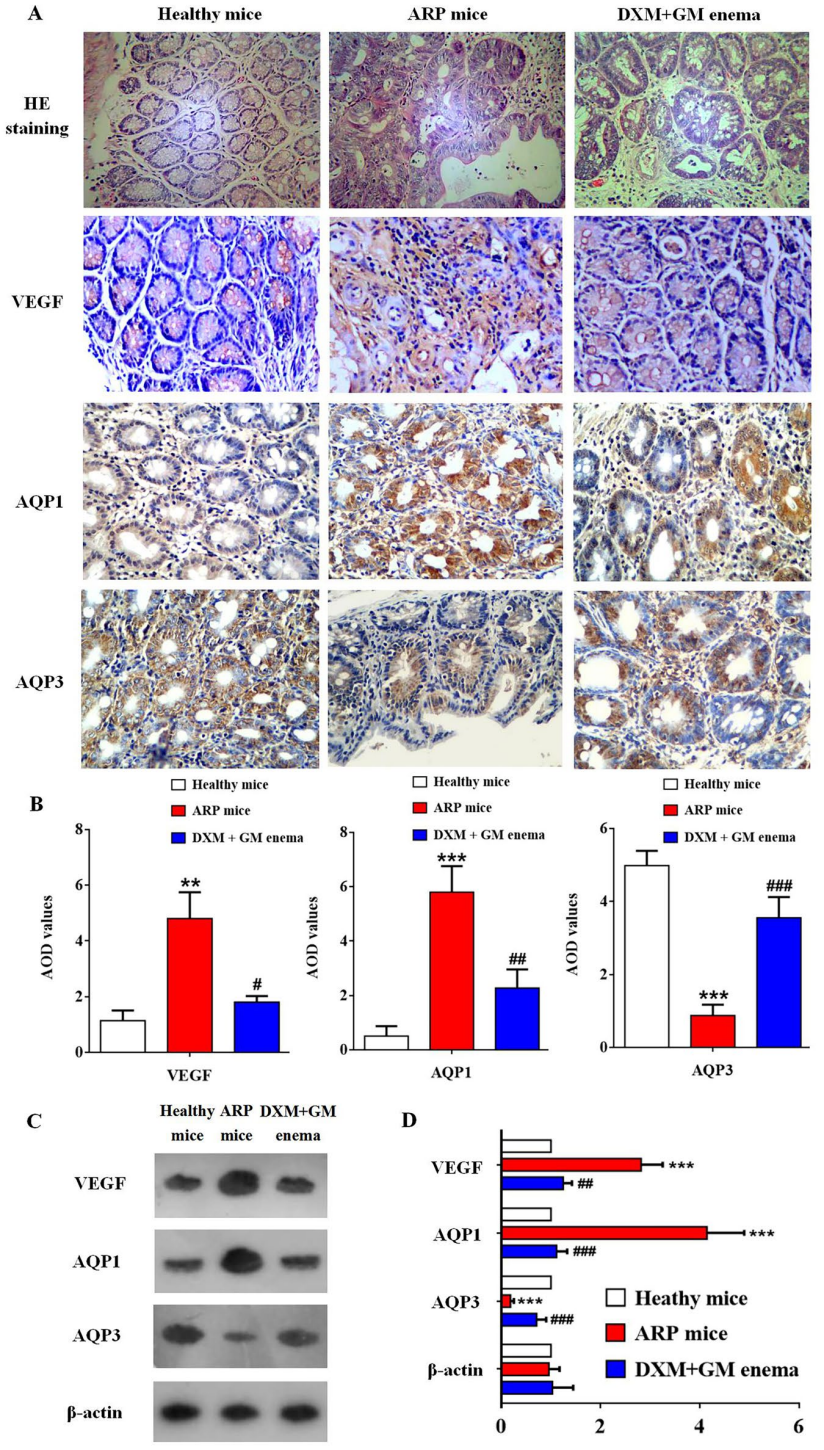
DXM + GM enema inhibits angiogenesis by downregulating VEGF and AQP1 and upregulating AQP3 in ARP mice. (**A**) The immunohistochemistry of VEGF, AQP1, AQP3 and HE staining in different groups of mice (n = 10 for each group) were observed under light microscopy (scale ×400). (**B**) AOD values of NF-κB were detected by immunohistochemistry in different groups of mice (**P < 0.01, ***P < 0.001, ^#^*P* < 0.05, ^##^*P* < 0.01, ^###^*P* < 0.001). (**C**) The expression of VEGF, AQP1 and AQP3 in different groups of mice was tested by using western blot. (**D**) The expression of VEGF and AQP1 in rectal tissue of ARP mice was significantly higher than that of healthy mice (***P < 0.001). However, the expression of AQP3 in rectal tissue of mice in ARP model group was decreased significantly compared to healthy control group (***P < 0.001). DXM + GM enema down-regulated VEGF and AQP1, while up-regulated AQP3 (^##^*P* < 0.01, ^###^*P* < 0.001).

As shown in Fig. 7, the expression of VEGF and AQP1 in rectal tissue of ARP mice was significantly higher than that of healthy mice. However, the expression of AQP3 in rectal tissue of mice in ARP model group was decreased significantly compared to healthy control group. DXM + GM enema down-regulated VEGF and AQP1, while up-regulated AQP3. These results suggested that the effect of AQP3 was contrary to the effect of AQP1 in the development of ARP.

### DXM + GM enema downregulates PI3K/AKT signaling pathway in ARP mice

**Figure 8:**
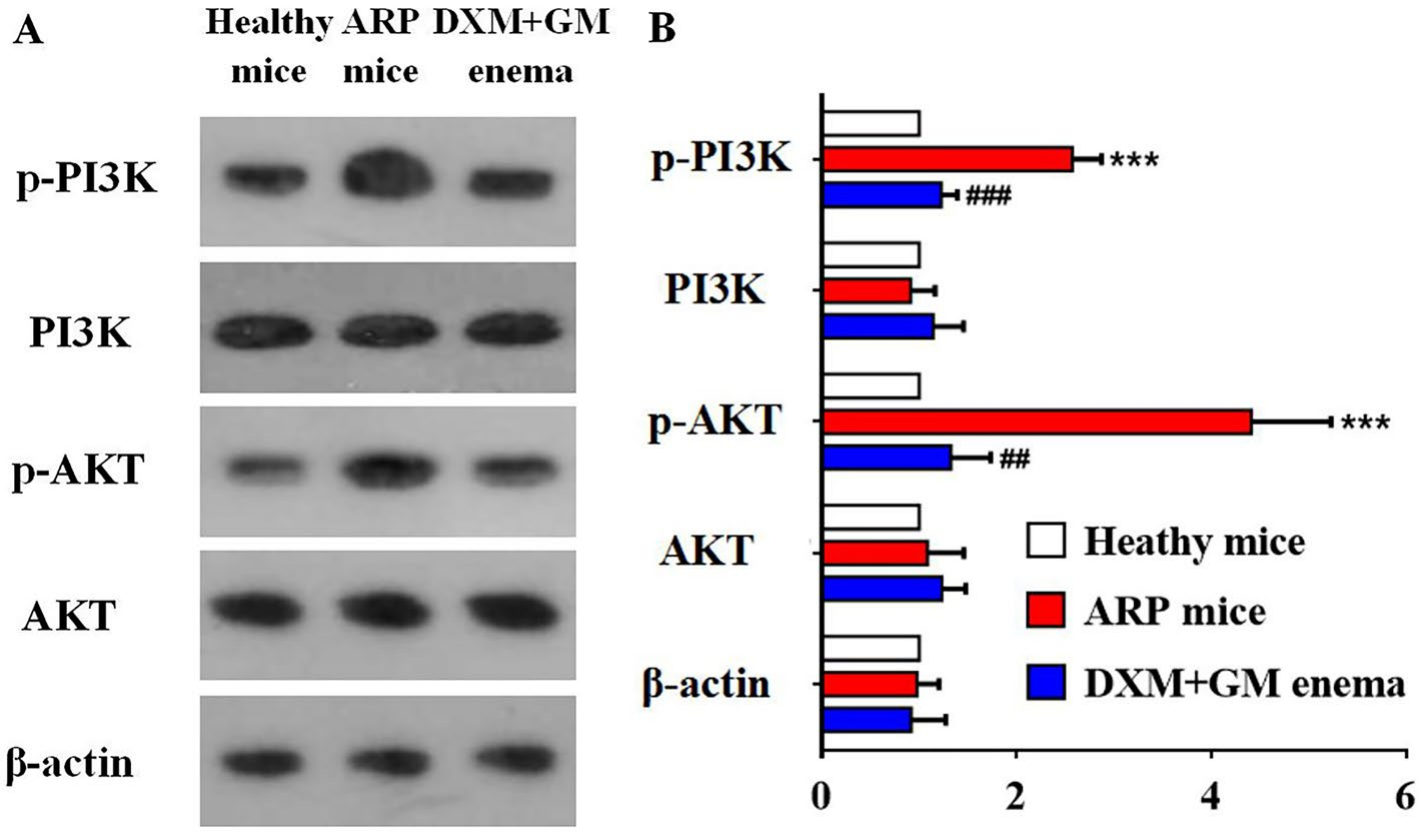
DXM + GM enema downregulates PI3K/AKT signaling pathway in ARP mice. (**A**) The expression of the PI3K/AKT signaling pathway was measured via western blot in rectal tissues of ARP mice treated with DXM + GM enema treatment. (**B**) The expression of p‑PI3K and p‑AKT in was increased in rectal tissues of ARP mice (***P < 0.001). However, the expression levels of p‑PI3K and p‑AKT were significantly decreased by DXM + GM enema treatment (^##^*P* < 0.01, ^###^*P* < 0.001).

To determine whether the PI3K/AKT signaling pathway was involved in inflammation-induced angiogenesis in ARP mice, the expression of the PI3K/AKT signaling pathway was measured via western blot in rectal tissues of ARP mice treated with DXM + GM enema (Fig. 8). The expression of p‑PI3K and p‑AKT in was increased in rectal tissues of ARP mice. However, the expression levels of p‑PI3K and p‑AKT were significantly decreased by DXM + GM enema treatment. These results indicated that DXM + GM enema downregulated the PI3K/AKT signaling pathway.

### DXM + GM enema leads to apoptosis in ARP mice

**Figure 9:**
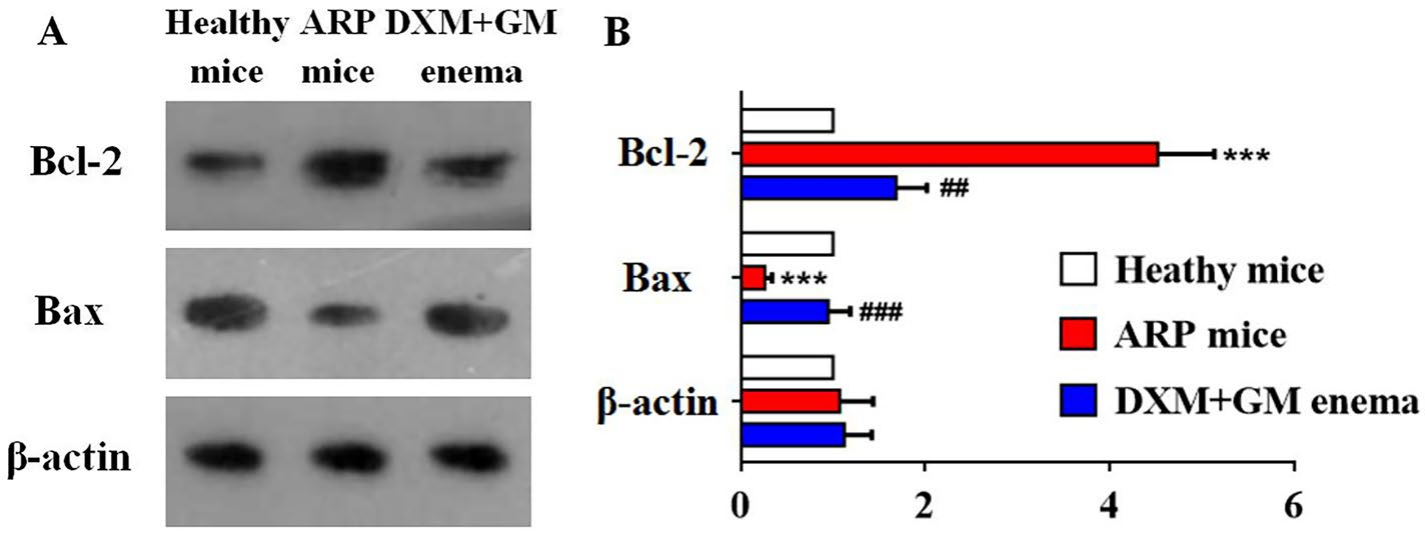
DXM + GM enema leads to apoptosis in ARP mice. (**A**) The expression levels of Bcl-2 and Bax protein in rectal tissues of ARP mice treated by DXM + GM enema using western blot. (**B**) The expression of Bcl-2 was increased while the expression of Bax was decreased in ARP mice (***P < 0.001). DXM + GM enema significantly decreased Bcl-2 expression and increased Bax expression in rectal tissues of ARP mice (^##^*P* < 0.05, ^###^*P* < 0.001).

We analyzed the effects of PI3K on Bax and Bcl-2 protein levels in rectal tissues of ARP mice treated by DXM + GM enema using western blot. DXM + GM enema significantly increased Bax expression and decreased Bcl-2 expression in rectal tissues of ARP mice (Fig. 9). These data demonstrated that PI3K might be correlated with the pro-apoptosis effects of DXM + GM enema on ARP.

### Novel mechanisms underlying inhibition of inflammation-induced angiogenesis by DXM + GM enema via PI3K/AKT/NF-κB/VEGF pathways in ARP mice

**Figure 10:**
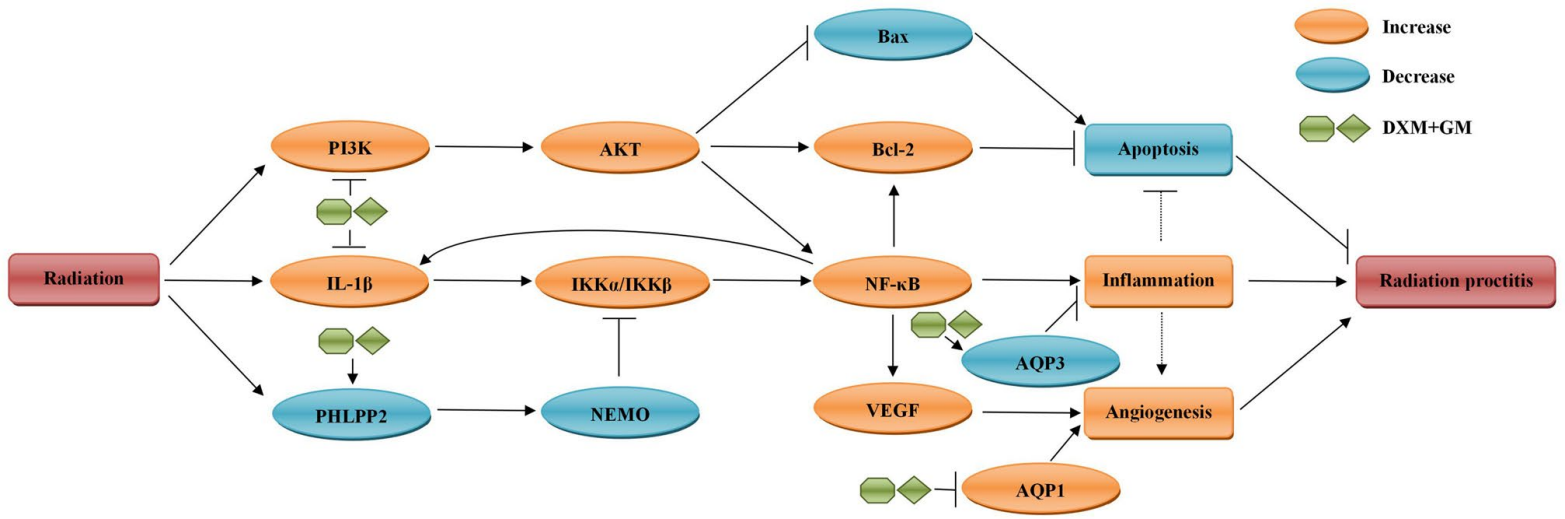
Novel mechanisms underlying inhibition of inflammation-induced angiogenesis by DXM + GM enema via PI3K/AKT/NF-κB/VEGF pathways in ARP mice. Radiation stimulates PI3K/AKT signaling pathway, which triggers intracellular signal cascade reaction, activates NF-κB and other pro-inflammatory cytokines. NF-κB promotes the transcription of IL-1β, VEGF and Bcl-2. IL-1β activates NF-κB and amplifies inflammatory. VEGF promotes angiogenesis and Bcl-2 exerts anti-apoptotic activity. Interestingly, DXM + GM enema exerts anti-inflammation and anti-angiogenesis by downregulating PI3K/AKT/NF-κB/VEGF and IL-1β/NF-κB signaling pathways and upregulating PHLPP2 and NEMO. Moreover, DXM + GM enema induced apoptosis by increasing Bax and suppressing Bcl-2 mediated by PI3K/AKT signaling pathway. Besides which, DXM + GM enema inhibits inflammation by upregulating AQP3 and inhibits angiogenesis by downregulating AQP1.

As shown in Fig. 10, we firstly found the mechanisms underlying inhibition of inflammation-induced angiogenesis by DXM + GM enema in ARP mice. Radiation stimulates PI3K/AKT signaling pathway, which triggers intracellular signal cascade reaction, activates NF-κB and other pro-inflammatory cytokines. NF-κB promotes the transcription of IL-1β, VEGF and Bcl-2. IL-1β activates NF-κB and amplifies inflammatory. VEGF promotes angiogenesis and Bcl-2 exerts anti-apoptotic activity. Interestingly, we found that DXM + GM enema exerted anti-inflammation and anti-angiogenesis by downregulating PI3K/AKT and IL-1β/NF-κB signaling pathways and upregulating PHLPP2 and NEMO. Moreover, DXM + GM enema induced apoptosis by increasing Bax and suppressing Bcl-2 mediated by PI3K/AKT signaling pathway. Besides which, DXM + GM enema inhibited inflammation by upregulating AQP3 and inhibited angiogenesis by downregulating AQP1.

## Discussion

In this study, we observed that differential genes regulated by DXM + GM enema inhibited ARP development, and the regulatory mechanism was evaluated. Results suggested that DXM + GM enema inhibited inflammation by downregulating NF-κB and upregulating AQP3, as well as inhibited angiogenesis by downregulating VEGF and AQP1 in ARP mice. Excitingly, the novel underlying mechanisms are clarified. DXM + GM enema inhibits the PI3K/AKT signaling pathway, which in turn leads to the downregulation of NF-κB and VEGF related signaling pathways. In addition, DXM + GM enema induces apoptosis by increasing Bax and suppressing Bcl-2 mediated by PI3K/AKT signaling pathway.

As the most common side-effect of pelvic radiotherapy, ARP is mainly characterized by diarrhea and bloody. These symptoms indicate that inflammation and angiogenesis are linked to the development of ARP^21,22^. Of two predominant pathologic changes, radiation-induced inflammation associated with diarrhea should be taken into the consideration as early pathologic change, and angiogenesis related with bloody may be regarded as late pathologic change of ARP. Radiation stimulates macrophages to produce a large number of IL-1β, which triggers intracellular signal cascade reaction, activates NF-κB and other pro-inflammatory cytokines. NF-κB promotes the transcription of IL-1β and amplifies inflammatory response^23^. Soon after inflammation, severe congestion and edema are found in rectal mucosa of ARP. Angiogenesis begins to participate in the pathological process of ARP. As everyone knows, VEGF is one of the most key cytokines in promoting angiogenesis^24^. Studies^5,21^ have demonstrated that the high expression of VEGF is related to angiogenesis in ARP. Here, we put forward a hypothesis that "inflammation-induced angiogenesis" exists in pathogenesis of ARP. The expression of VEGF may be upregulated by NF-kB accompanying the activation of IL-1β. In order to explore the specific mechanisms underlying the inflammation-induced angiogenesis, the present study analyzed global gene expression in rectal mucosa from ARP mice.

Based on transcriptome sequencing data of ARP mice, we found that *Mmp7*, *Hp*, *Saa3*, *Slpi*, *Ceacam12* and *Pla2g2a* were significantly increased, while *Il13ra2*, *Ttc7* and *Gsdmc* were significantly decreased in ARP mice. These genes are related to not only inflammation but also angiogenesis. Some genes promote inflammatory and or angiogenesis, such as *Mmp7*, *Hp*, and *Saa3*. For example, MMP7 plays a critical role in inflammation and angiogenesis by inducing extracellular matrix degradation. HP is an inflammation-inducible plasma protein^25^, and also has potent angiogenesis^26^. SAA3 is an acute phase systemic inflammation marker^27^, which can induce angiogenesis^28^. Other genes exert anti-inflammatory effect, such as *Slpi*, *Ceacam12*, *Pla2g2a*, *Il13ra2*, *Ttc7* and *Gsdmc*. SLPI is known to inhibit the inflammatory response in tissue repair^29^. CEACAM1 negatively regulates inflammation in inflammatory bowel disease models^30^. PLA2G2A contributes to the low grade inflammation associated with hypothyroidism^31^. The IL-13 is a key suppressive inflammation mediator regulated by IL-13Rα2^32^. TTC7A deficiency leads to intestinal abnormality and inflammation^33^. GSDMC is a crucial mediator of pyroptosis downstream of and inflammatory cascade activation^34^. These results suggest that ARP progression is orchestrated by the complex balance between pro-inflammation factors and anti-inflammation factors. And inflammation-induced angiogenesis is the key mechanism in the pathological progress of ARP. After treatment of DXM + GM enema, *Ttc7*, *Phlpp2*, *Ikbkg* (NEMO), *Gsdmc* and *Sumo3* were upregulated, while *Egr-2* and *Bak1* were significantly downregulated. PHLPP2 loss enhances NEMO ubiquitination and subsequent IKKβ phosphorylation, resulting in increased NF-κB-dependent transcription of multiple target genes^35^. Our data showed that *Phlpp2* and NEMO were upregulated in ARP mice treated by DXM + GM enema. DXM + GM enema might suppress NF-κB pathway by upregulating genes including *Phlpp2* and NEMO, but the underlying mechanisms remain to be clarified (Supplementary Information).

Activation of the PI3K/AKT signaling pathway in response to radiation has been widely observed in some studies^36,37^. PI3K/AKT signaling pathway plays a vital role in inflammation^38,39^, angiogenesis^40^, proliferation^41^ and apoptosis^42^. By catalyzing the phosphorylation of a series of proteins, the activated AKT induces inflammation, enhances angiogenesis, promotes proliferation and inhibits apoptosis. Upon activation, AKT induces expression of NF-κB^43^, which promotes the transcription of a wide range of genes^44^. NF-κB is a type of heterodimer composed of a p50 and a p65 subunit, which forms a compound with its inhibitor IκB under normal conditions^45^. Following stimulation of radiation, IκB in NF-κB trimer is phosphorylated, then ubiquitinated and degraded, thus leading to its dissociation with NF-κB. Subsequently, NF-κB is activated, enters the cell nucleus, and induces the transcription of VEGF, Bcl-2 and Bcl-xL^46^. Of note, IκB needs NEMO serves as a gatekeeper for its phosphorylation^47^. NEMO, IKKα and IKKβ compose a complex, which is essential for NF-κB activation^48^. NEMO binds ubiquitin directly and allows activation of the IKKs. IKKs mediate IκB phosphorylation and subsequent degradation, and then lead to NF-κB activation. In the current in vivo study, we found that PI3K/AKT signaling pathway was inhibited whereas PHLPP2 and NEMO were upregulated by DXM + GM enema. These results indicate that DXM + GM enema ameliorates ARP by inhibiting PI3K/AKT/NF-κB/VEGF signaling pathways. The mechanisms are related to increase of PHLPP2 levels and subsequent decreased ubiquitination of NEMO, which inhibits IκB phosphorylation mediated by IKKα/IKKβ phosphorylations for an optimal NF-κB inhibition. In addition, there is a positive feedback loop between VEGF and PI3K/AKT signaling pathway. VEGF triggers angiogenesis through PI3K/AKT signaling pathway, which increases NF-κB dependent VEGF transcription. DXM is known to interact with NF-κB via the glucocorticoid receptor (GR). There are well-known antagonistic interactions between NF-κB and GR. In PANC-1 human pancreatic cancer cells expressing abundant GR, NF-κB phosphorylation and VEGF was significantly downregulated by DXM^49^. However, the role of GM in the regulation of NF-κB and VEGF still needs to be researched.

Besides VEGF, endothelial cell migration mediated by aquaporin also plays important roles in angiogenesis^50^. Aquaporins are a class of highly selective transmembrane channels that allow transmembrane transport of water and some small solute molecules^51^. Up to now, 13 subtypes of aquaporins have been found in mammals^52^. AQP1 is an important highly selective water transport channel^53^. AQP1 has been found to be highly expressed in many tumors^54^, which is positively correlated with microvessel density, promotes endothelial cell migration, and is involved in angiogenesis^55^. During the process of ARP, AQP1 may induce angiogenesis by regulating the transmembrane transport of water and promoting the migration of endothelial cells. Our study found that the expression of AQP1 in ARP mice was increased, and inhibited by DXM + GM enema. Different from the high selectivity of AQP1 to water, AQP3 not only transports water, but also transports small molecules such as glycerol and urea^56^. AQP3 is an important regulator of intestinal water metabolism, secretion and absorption^57^. AQP3 knockout mice given dextran sulphate developed more severe colitis compared to wild-type mice^58^. It was found that the expression of AQP3 was decreased in intestinal inflammation induced by trinitrobenzene sulfonic acid, which reduced the intestinal water re-absorption and resulted in diarrhea symptoms^59^. Our results showed that the expression of AQP3 in ARP mice was significantly decreased, and increased by DXM + GM enema. Interestingly, during the pathological changes of inflammation-induced angiogenesis in ARP, AQP1 and VEGF promotes angiogenesis, while AQP3 inhibits inflammation.

PI3K/AKT signaling pathway also regulates anti-apoptotic pathways^60^. Phosphorylated AKT inhibits a number of pro-apoptotic factors including Bad, Bax and Bim^61^. Moreover, phosphorylated AKT induces activation of NF-κB, which promotes the transcription of anti-apoptotic genes, in particular Bcl-2 and Bcl-xL^61^. Bcl-2 can form a heterodimer with the pro-apoptotic factor Bax, which inhibits cell apoptosis. Our results indicated that DXM + GM enema significantly inhibited the PI3K/Akt pathway, then increased Bax and suppressed Bcl-2, which promoted cell apoptosis in rectal tissue of ARP mice. DXM + GM enema also decreased spleen weight, but did not affect liver weight. Spleen is the largest lymphoid organ^62^ and modulates the immune system by producing lymphocytes, immunoglobulin and complement, etc. Our previous study found that DXM + GM enema reduced spleen weight and promoted lymphocyte apoptosis by increasing Bax and suppressing Bcl-2^5^. We propose that apoptosis induction of DXM + GM may be an organ-specific effect. The high-dose DXM decreases the spleen weight of rat^63^. DXM may induce apoptosis in spleen, thereby reducing the spleen weight and exerting its immunosuppressive effect. However, the specific mechanisms such as apoptosis induction of GM need further study. Contrary to spleen weight, mice body weight was increased in DXM + GM enema treatment group compared to healthy control group. The side effects of DXM are known as sodium retention, obesity and high blood pressure. We consider that more weight gain in the DXM + GM group may be a side effect of DXM, which is beneficial for the treatment of ARP in the short term.

However, there were several questions still remain unanswered. Firstly, considering the treatment of the combination of DXM and GM was used in patients with ARP and ARP mice model for many years in China, we focus on the effect of the combination of DXM and GM in ARP mice, not distinguish the different effects and roles of DXM and GM alone in present study. More research is needed to evaluate the effects of DXM and GM alone and in combination in ARP mice and healthy mice, and elucidate their underlying mechanisms. Secondly, DXM + GM enema induced the apoptosis in spleen and rectum, but not in other organs. The apoptosis induction of DXM + GM may be an organ-specific effect. The specific mechanisms of apoptosis induced by DXM alone and in combination with GM need further study. Thirdly, DXM is known to interact with NF-κB via GR. NF-κB and GR may antagonize each other's activity. Whether the interaction between DXM and NF-κB via the GR is related to the effect of DXM + GM, which is still an important issue. Finally, DXM induces a DNA repair gene O6-methylguanine-DNA methyltransferase (MGMT). Further research is needed to clarify whether DXM + GM enema affects the DNA damage or repair of irradiated cells and tissues caused by radiation.

In conclusion, we firstly demonstrate that the anti-inflammation, anti-angiogenesis and apoptosis induction effects of DXM + GM enema on ARP are mainly mediated through the PI3K/AKT/NF-κB/VEGF signaling pathways (Fig. 10). These findings indicate that DXM + GM enema inhibits inflammation-induced angiogenesis in ARP and may provide a novel target for ARP treatment.

## Materials and methods

### Mice

5–7 weeks old, 18 ± 2 g weight and healthy C57BL/6J mice of both sexes, were obtained from Beijing Haidian Xingwang experimental animal farm (license number: SCXK-2014-0013). The mice were raised under specific-pathogen-free conditions and were free to diet and water for one week in the central laboratory of Shanxi province research institute of traditional Chinese medicine. The laboratory temperature was 22 ± 3 degrees centigrade and the relative humidity was 55 ± 10%. The mice identified by earmarks were randomly divided into three group (10 mice per group), including healthy control group (distilled water enema), ARP model group (distilled water enema), and DXM + GM enema treatment group (DXM 0.83 mg/kg + GM 13,300 U/kg). All methods were performed in accordance with relevant guidelines and regulations. All procedures were approved by the ethics committee on animal experiments of Shanxi province research institute of traditional Chinese medicine (20150318) and conformed to ARRIVE guidelines 2.0 published in PLOS Biology.

### Ethical approval and informed consent

All methods were performed in the central laboratory of Shanxi province research institute of traditional Chinese medicine and were carried out in accordance with relevant guidelines and regulations. All animal experiments were approved by the ethics committee on animal experiments of Shanxi province research institute of traditional Chinese medicine (20150318). All methods are reported in accordance with ARRIVE guidelines 2.0 for the reporting of animal experiments.

### Laboratory medicine and antibody

Dexamethasone sodium phosphate injection (specification: 1 ml: 5 mg, license number: NMPN H37021969) was purchased from Chenxin Pharmaceutical Incorporated Company. Gentamicin sulphate injection (specifications: 2 ml: 8 million units, license number: NMPN41025466) was purchased from Henan Furen Huaiqingtang Pharmaceutical Company Limited. Anti-p-PI3K (cat. no. 4228), anti-PI3K (cat. no. 4292), anti-p-AKT (cat. no. 4060), anti-AKT (cat. no. 4691), anti-p-TAK1 (cat. no. 9339), anti-TAK1 (cat. no. 4505), anti-p-IKKα/β (cat. no. 2697), anti-IKKα (cat. no. 11930), anti-IKKβ (cat. no. 2678), anti-p-IκBα (cat. no. 2859), anti-IκBα (cat. no. 4814) were purchased from Cell Signaling Technology, Inc. Anti-IL-1β (sc-12742), anti-VEGF (sc-7269), anti-AQP1 (sc-25287), anti-AQP3 (sc-518001), anti-Bcl-2 (sc-7382), anti-Bax (sc-7480), and β-actin (sc-47778) were purchased from Santa Cruz Biotechnology. Anti-NF-κB (ab16502), anti-PHLPP2 (ab71973) and anti-NEMO (ab178872) was purchased from ABCAM. Horseradish peroxidase (HRP)-conjugated goat anti-mouse IgG (H + L) (cat. no. AP308P) and unconjugated goat anti-rabbit IgG (cat. no. AP132) were purchased from Sigma-Aldrich (Merck KGaA). DAB concentrated kits (PAB180021) was purchased from Bioswamp.

### ARP mice model

Although fractionated irradiation is consistent with the characteristics of clinical radiotherapy, it is difficult to carry out related experiments because of high early mortality in mice^64^. Similar to previous studies^65,66^, ARP mice were established by a single large dose local radiation of pelvic cavity. Mice of ARP model group and DXM + GM enema treatment group were anesthetized by intraperitoneal injection with pentobarbital sodium (50 mg/kg), and then they were fixed in the supine position and irradiated with 27 Gy at a dose rate of 400 cGy/min 6 MV X-ray by using linear accelerator (CX Series, Varian Company, USA). Lead shielding (5 half value layer) was used to cover the mice except for a 1 cm × 1.3 cm area from the pubic symphysis to the anus in the middle of the field. This model of localized single-dose radiation exposure does not simulate fractionation treatment, but actually generates histopathological lesions similar to those seen clinically (ie, severe acute mucosal ulceration and transmural collagen deposition during the late phase)^67,68^.

### Enema method

Mice in different groups were treated by distilled water or DXM + GM enema 1 time a day for 2 weeks. After treatment for 2 weeks, mice were sacrificed after anesthesia and 1 cm tissues of upper rectums were collected. These rectum tissues were divided into two segments. Half tissue segments were placed in an EP tube and stored in a refrigerator at − 80 °C for western blot. The other tissue segments were fixed with paraffin embedded for H&E staining and immunohistochemistry staining.

### Evaluation of general signs of mice

The general signs of mice were scored as follows^69^: (1) formed stool, intact anal skin, normal diet and water intake, no weight loss, normal activity, and smooth and clean hair; (2) unformed mucous stool, the longest diameter of perianal hair loss region was < 0.5 cm, slightly decreased diet and water intake, 1–2 g weight loss, normal activity, and slightly worse hair; (3) diarrhea, mucous bloody stool, the longest diameter of perianal hair loss region was 0.5–1 cm, significantly decreased diet and water intake, 2–3 g weight loss, curled up and occasionally less movement, and significantly worse hair; (4) diarrhea, watery stool and (or) bloody stool, the longest diameter of perianal hair loss region was > 1 cm, anorexia, weight loss was > 3 g, curled up and less movement, and significantly worse hair.

### Pathological grade of rectal tissue

Pathological changes of rectal tissue were graded as follows^70^: Grade 0, normal rectal mucosa or minor pathological changes which are uncorrelated with radiation; Grade 1, slight damage characterized by slight inflammation and/or crypt change; Grade 2, mild damage, such as mild inflammation and/or crypt change; Grade 3, moderate damage, such as obvious inflammation and/or rectal epithelium loss; and Grade 4, serious damage presented as rectal ulceration or necrosis.

### Spleen index and liver index

After treatment for 2 weeks, the mice were weighed and anesthetized by intraperitoneal injection of pentobarbital sodium (50 mg/kg), and then they were sacrificed by cervical dislocation. Afterward, livers and spleens of mice were collected and weighed. The liver index was calculated using the following formula: liver index (%) = liver wet weight (g)/mouse body weight (g) × 100%. The spleen index was calculated using the following formula: spleen index (%) = spleen wet weight (g)/mouse body weight (g) × 100%.

### Transcriptome sequencing

Total RNA of rectum was extracted with Trizol reagent before and after treatment of DXM and GM combination enema for 2 weeks. RNA purity was determined by using Nanodrop spectrophotometer. RNA quality was tested by Agilent 2100 Bioanalyzer. Purified RNA was used for cDNA synthesis and library preparation, and was sequenced on Illumina Second-generation DNA sequencing platform. Then, PCR products with a fragment size of 300–500 bp were sequenced in the Illumina DNA sequencing platform with a read length of 150 bp. The differentially expressed genes among different groups were identified with the HISAT2, stringtie and DESeq. Molecular function, biological process and cellular component were analyzed by using Gene ontology (GO, http://www.geneontology.org). The main physiological or biochemical metabolisms were identified by KEGG pathway analysis^71–73^ (Kyoto Encyclopedia of Genes and Genomes, http://www.kegg.jp).

### Western blot analysis

After enema treatment for 2 weeks, mice were sacrificed after anesthesia. Rectums were collected and stored in a refrigerator at − 80 °C. Then rectal tissues were lysed by protein lysis buffer (10 mM triethanolamine at pH 7.6 and 250 mM sucrose) and protease inhibitor cocktail (1 mM PMSF, 20 mM NaF and 1 mM Na_3_VO_4_). The rectal cell lysates were centrifuged at 13,000 rpm for 20 min at 4 °C. Protein concentrations were determined by using the BCA protein assay. Proteins (28 µg/lane) were loaded on a SDS-gel, and separated by SDS-PAGE and transferred to PVDF membrane. Different antigen bands on PVDF membranes were cut based on the molecular size marker before hybridization with different kinds of antibodies when needed. 5% skimmed milk was used to block nonspecific binding at room temperature for 90 min. PVDF membranes were incubated overnight at 4 °C with different primary antibodies at dilutions (anti-p-PI3K 1:1,000, anti-PI3K 1:1,000, anti-p-AKT 1:1,000, anti-AKT 1:1,000, anti-p-TAK1 1:1,500, anti-TAK1 1:1,000, anti-p-IKKα/β 1:1,500, anti-IKKα 1:1,000, anti-IKKβ 1:1,000, anti-p-IκBα 1:1500, anti-IκBα 1:1500, anti-IL-1β 1:2,000, anti-VEGF 1:1,000, anti-AQP1 1:1,000, anti-AQP3 1:1,000, anti-Bcl-2 1:1500, anti-Bax 1:1500, β-actin 1:1,000, anti-NF-κB 1:1,000, anti-PHLPP2 1:2,000, anti-NEMO 1:3,000). Then PVDF membranes were washed with 0.1% TBST and incubated with secondary antibodies at room temperature for 40 min. The specific protein bands were visualized by using ECL chemiluminescence detection kit and quantified by Quantity One software.

### Immunohistochemistry

Rectal tissue were fixed in 4% formaldehyde, dehydrated, embedded in paraffin, and stained with H&E. The tissue section was blocked by 1% hydrogen peroxide in methanol for 10 min. Antigen was retrieved by microwave in 10 mM citrate buffer (pH 6.0) at 95 °C for 10 min. Section slides were blocked with goat serum for 20 min to reduce nonspecific binding, and incubated in a humidified chamber at 4 °C overnight with different primary antibodies at dilutions (anti-NF-κB 1:100, anti-VEGF 1:100, anti-AQP1 1:100, and anti-AQP3 1:100). Then slides were washed three times with phosphate-buffered saline (PBS), and incubated for 20 min with biotinylated second antibody, and labeled with horseradish peroxidase. Peroxidase activity was observed blindly by two experienced pathologists. The positive staining was quantitatively analyzed by Image-Pro-plus software, and the average optical density (AOD) was the result of cumulative optical density (IOD) divided by total area (Area). These results were statistical analyzed.

### Statistical analysis

The experimental data were analyzed by statistical software SPSS16.0. The measurement data are expressed in mean ± SEM. One way ANOVA method was used for the comparison of homogeneity of variance among multiple groups. Dunnett T3 method was used for multiple comparisons of heterogeneity of variance. The rank sum test was used for the evaluation of pathological grade. *P* < 0.05 was considered to indicate a statistically significant difference. Figures were made with GraphPad Software Prism version 6.01 for Windows (GraphPad Software, San Diego, Calif., www.graphpad.com).

## Supplementary Information

Supplementary Information.

## Data Availability

The datasets generated during the current study are available from the corresponding author on reasonable request.

## Abbreviations

ARP: Acute radiation proctitis
CRP: Chronic radiation proctitis
DXM: Dexamethasone
GM: Gentamicin
NF-κB: Nuclear factor-kappa B
VEGF: Vascular endothelial growth factor
GO: Gene ontology
KEGG: Kyoto encyclopedia of genes and genomes
Phlpp2: PH domain and leucine-rich repeat protein phosphatase 2
NEMO: NF-kB essential modulator
