# Supplementary material for: Novel mechanisms underlying inhibition of inflammation-induced angiogenesis by dexamethasone and gentamicin via PI3K/AKT/NF-κB/VEGF pathways in acute radiation proctitis
Source: Sci Rep. 2022 Aug 18;12:14116. doi: 10.1038/s41598-022-17981-8 (PMC9388498; doi:10.1038/s41598-022-17981-8)

**Figure S1.** ***DXM + GM enema ameliorates inflammation by inhibiting NF-κB signaling pathway in ARP mice*.** The expression of NF-κB signaling pathway was tested by using western blot. β-actin was used as loading internal control. The original images shown derive from the triplicate experiments. The list as followed as healthy mice, ARP mice and DXM + GM enema. Membranes were cut based on the size marker and hybridized with different kinds of antibodies when needed. The red boxes indicate the cropped areas shown in Figure 6B.


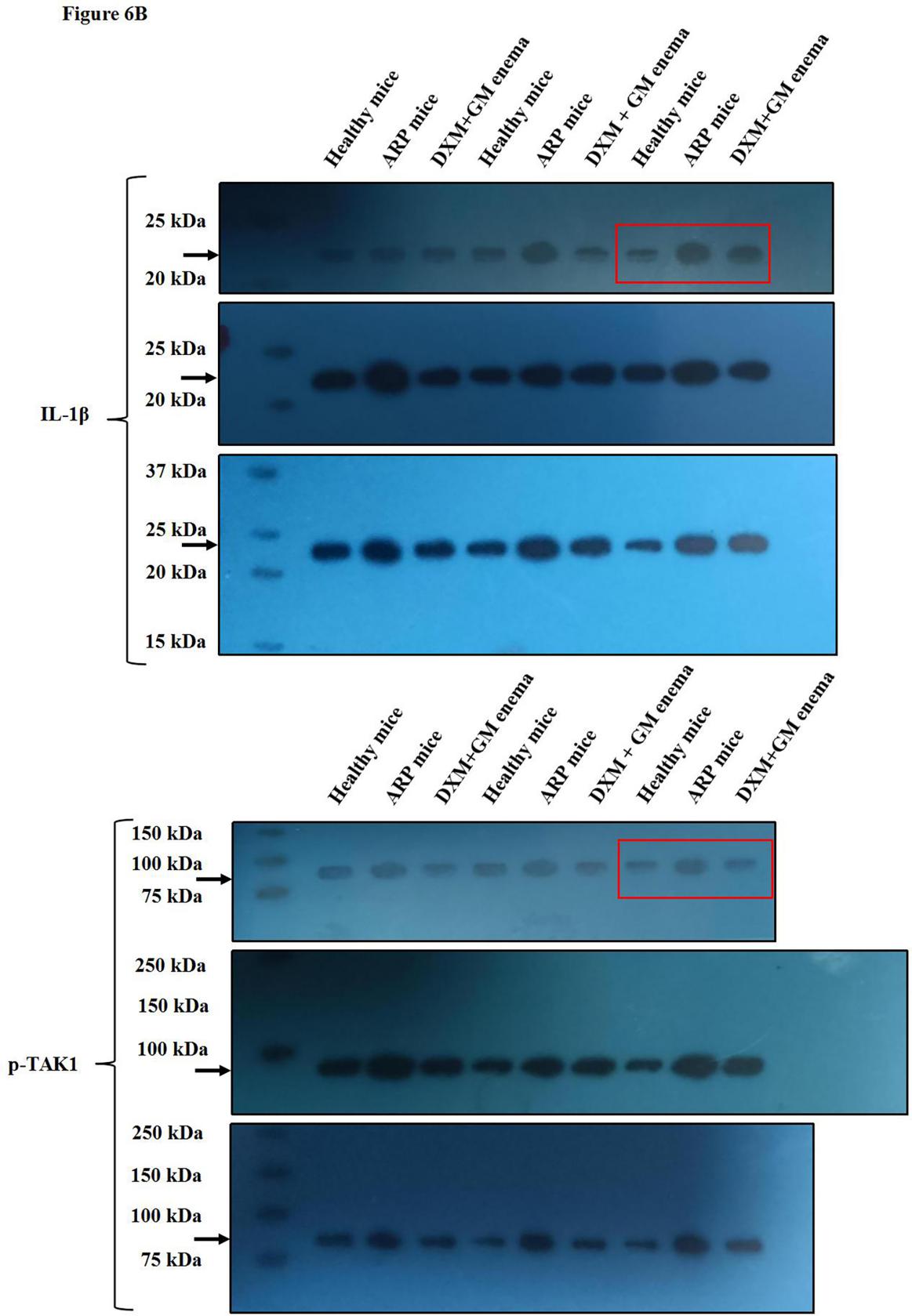


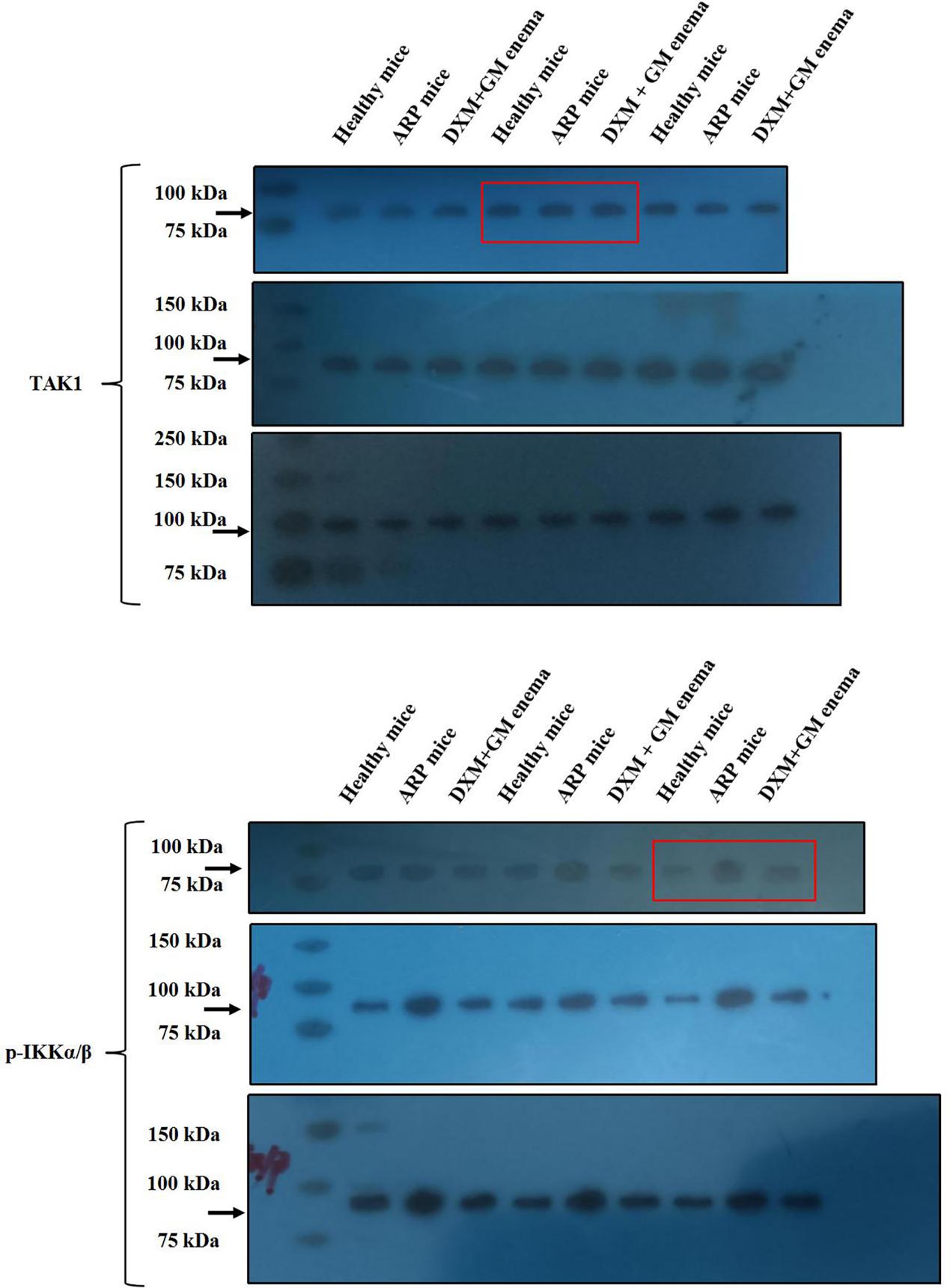


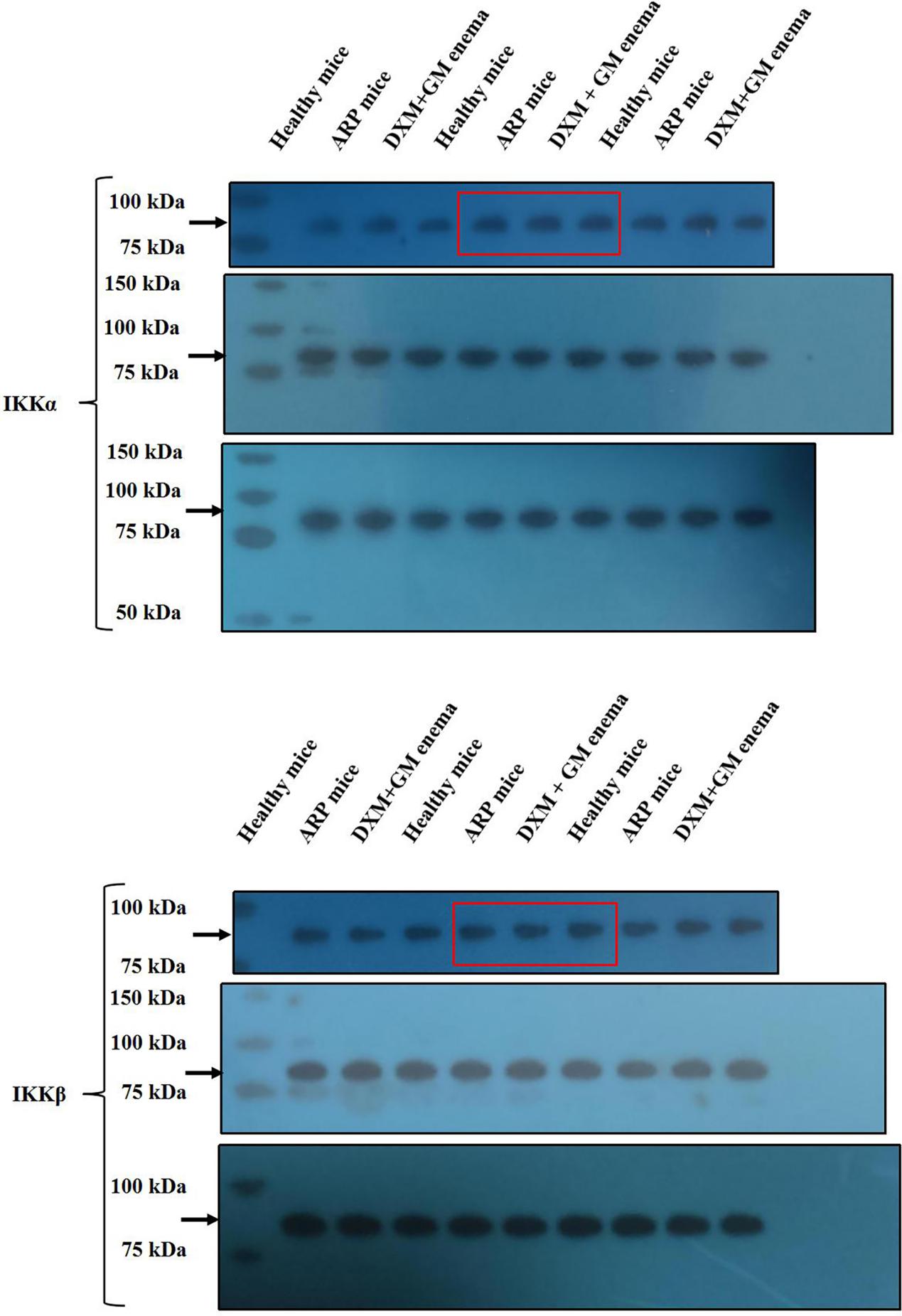

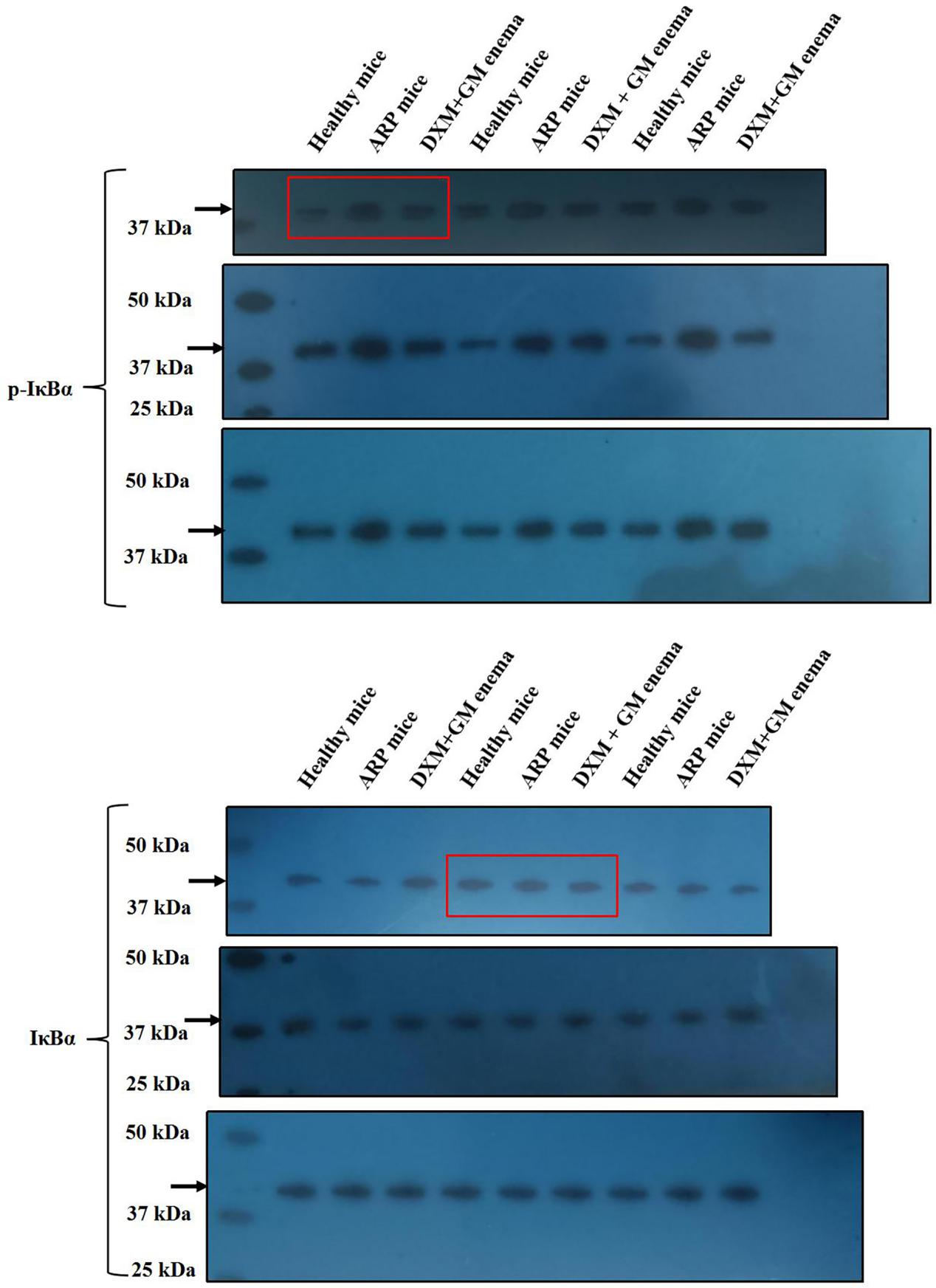

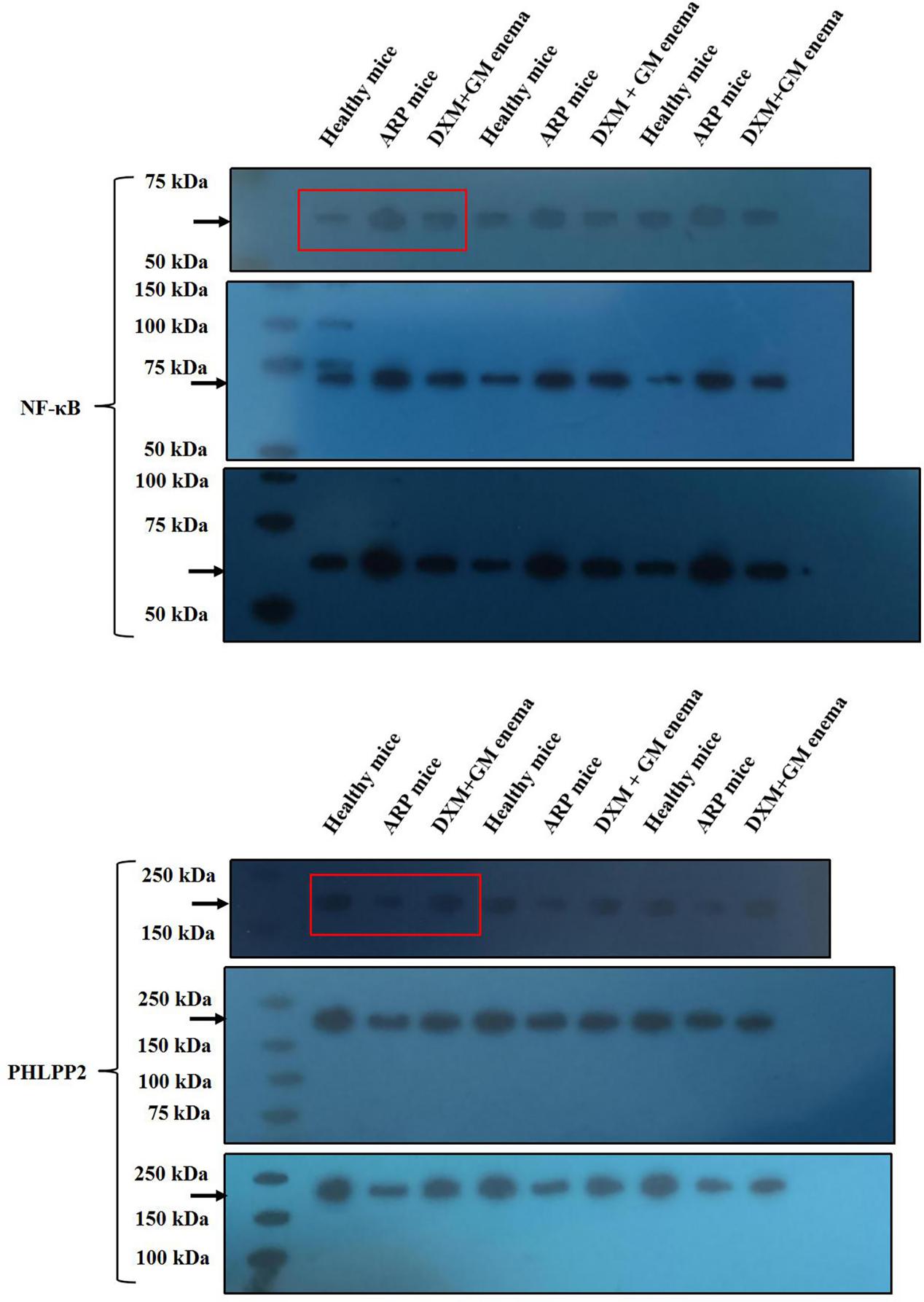

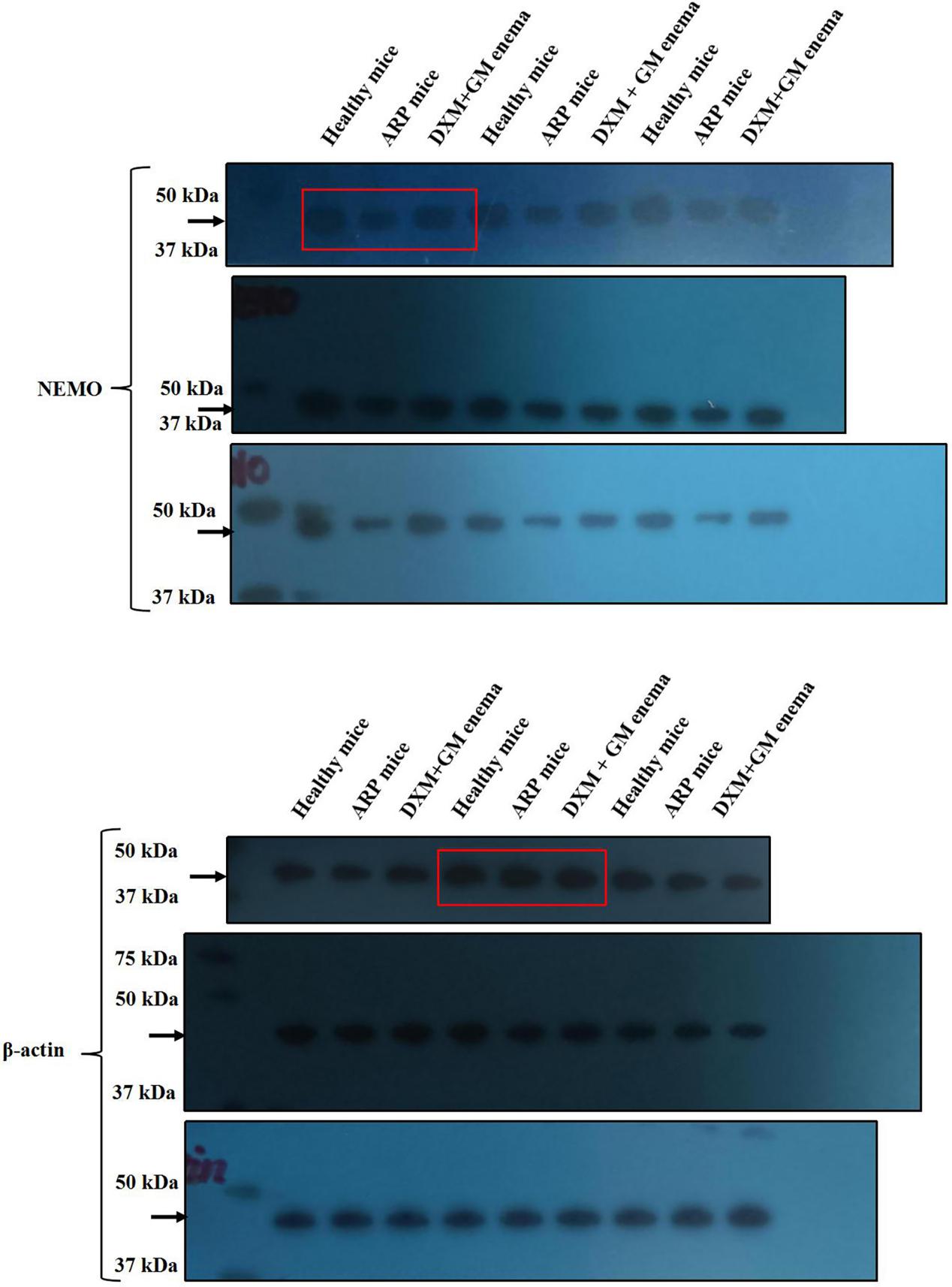


**Figure S2.** ***DXM + GM enema inhibits angiogenesis by downregulating VEGF and AQP1 and upregulating AQP3 in ARP mice*.** The expression of VEGF, AQP1 and AQP3 in different groups of mice was tested by using western blot. β-actin was used as loading internal control. The original images shown derive from the triplicate experiments. The list as followed as healthy mice, ARP mice and DXM + GM enema. Membranes were cut based on the size marker and hybridized with different kinds of antibodies when needed. The red boxes indicate the cropped areas shown in Figure 7C.


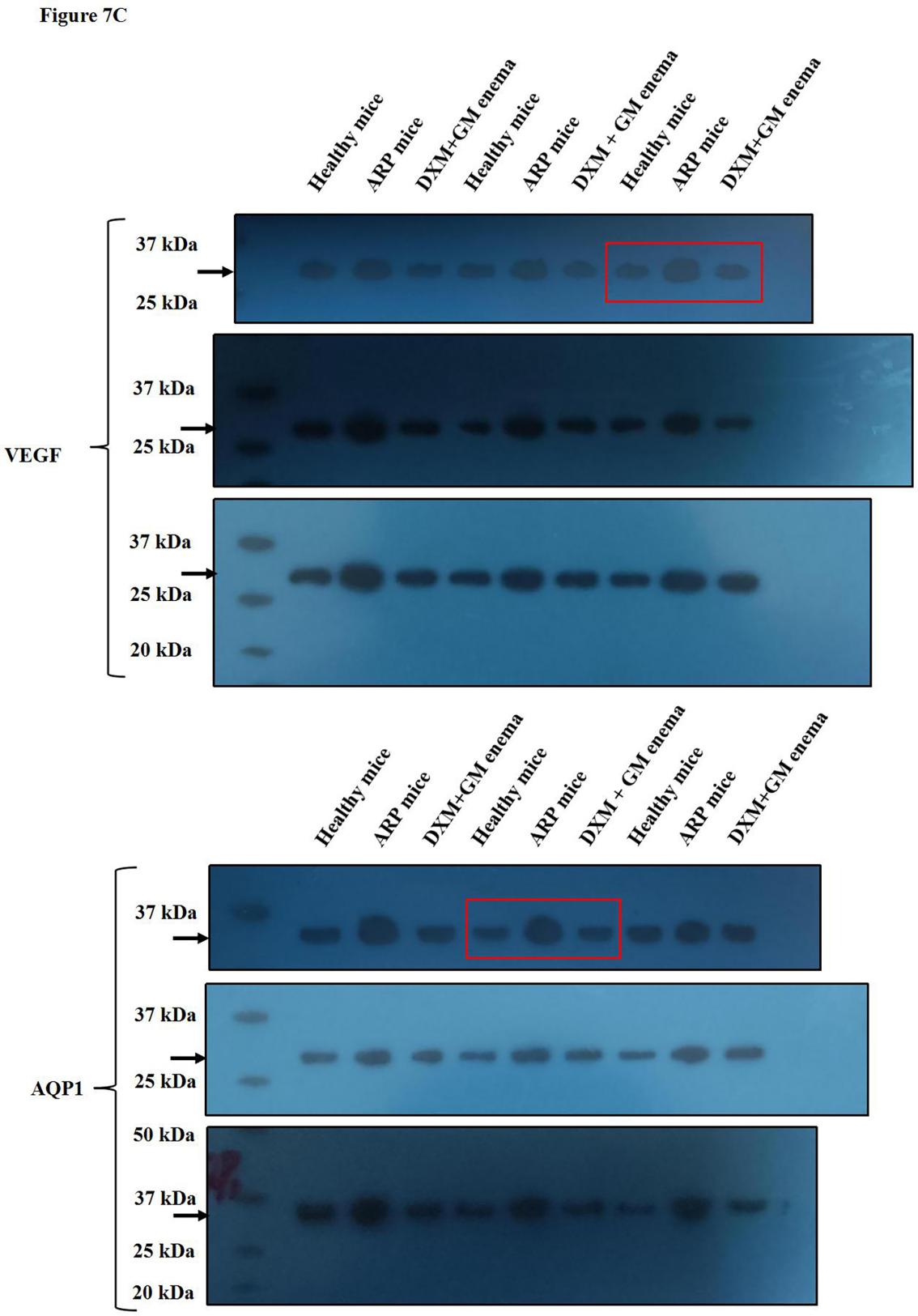


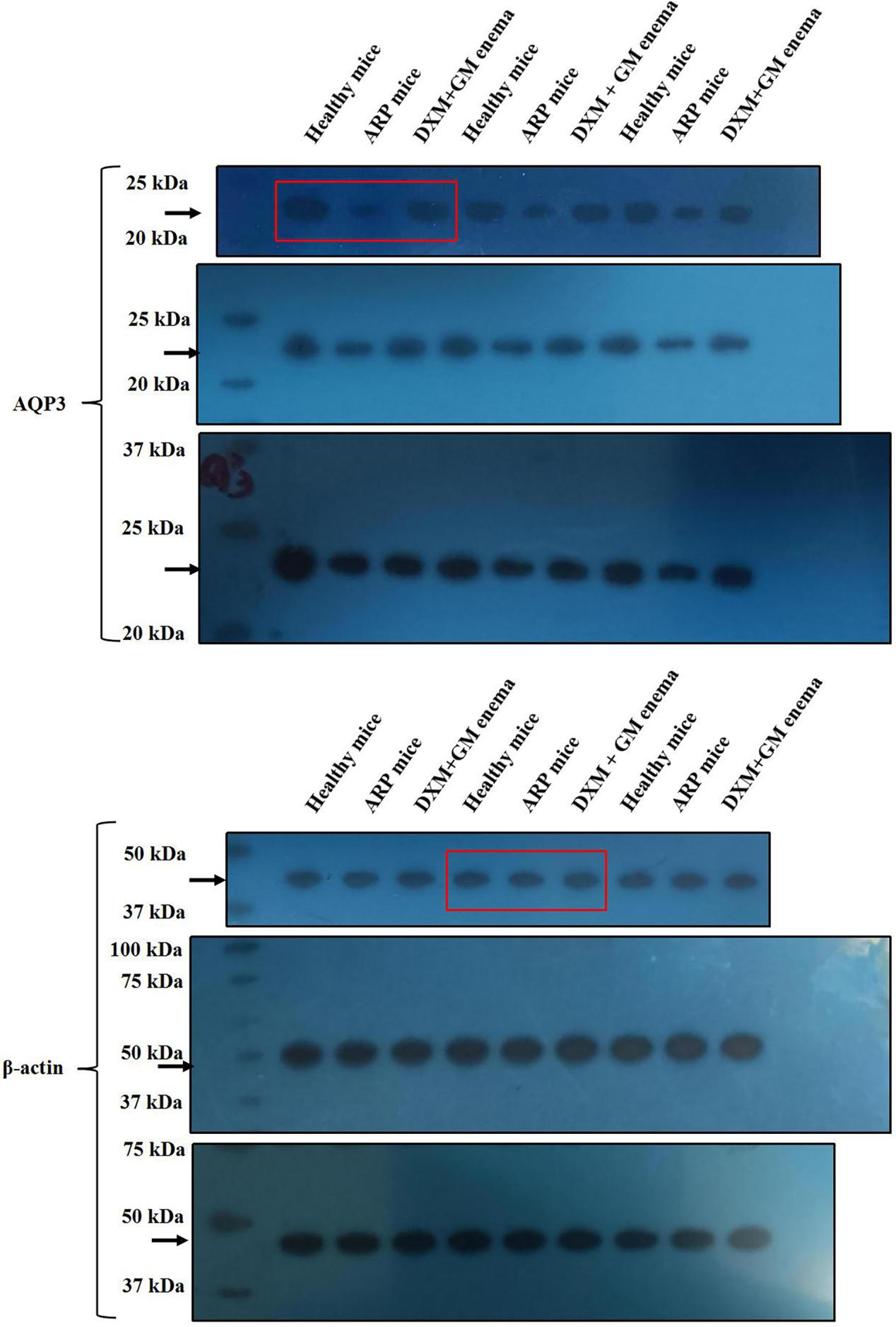


**Figure S3.** ***DXM + GM enema downregulates PI3K/AKT signaling pathway in ARP mice*.** The expression of the PI3K/AKT signaling pathway was measured via western blotting in rectal tissues of ARP mice treated with DXM + GM enema treatment. β-actin was used as loading internal control. The original images shown derive from the triplicate experiments. The list as followed as healthy mice, ARP mice and DXM + GM enema. Membranes were cut based on the size marker and hybridized with different kinds of antibodies when needed. The red boxes indicate the cropped areas shown in Figure 8A.


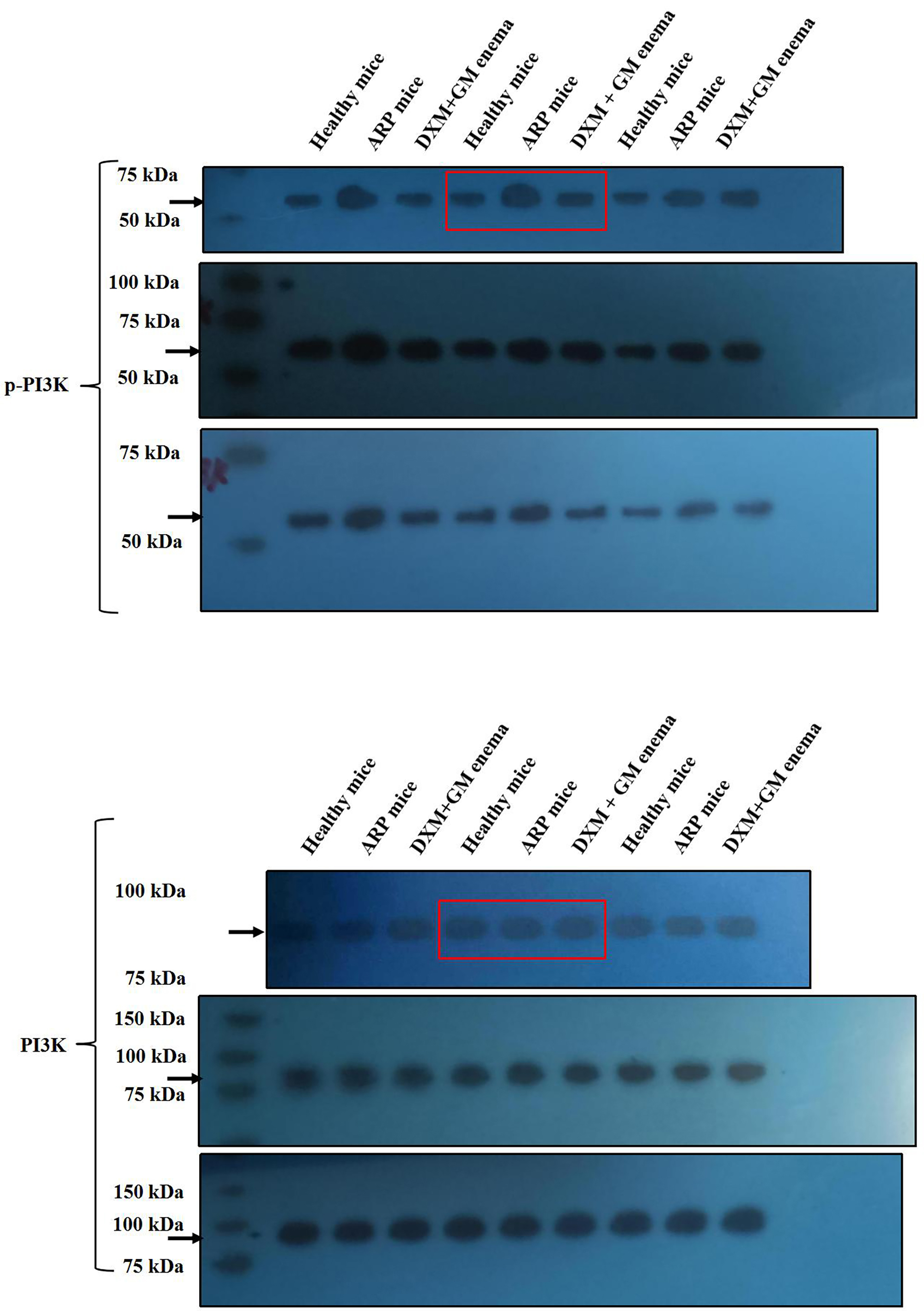


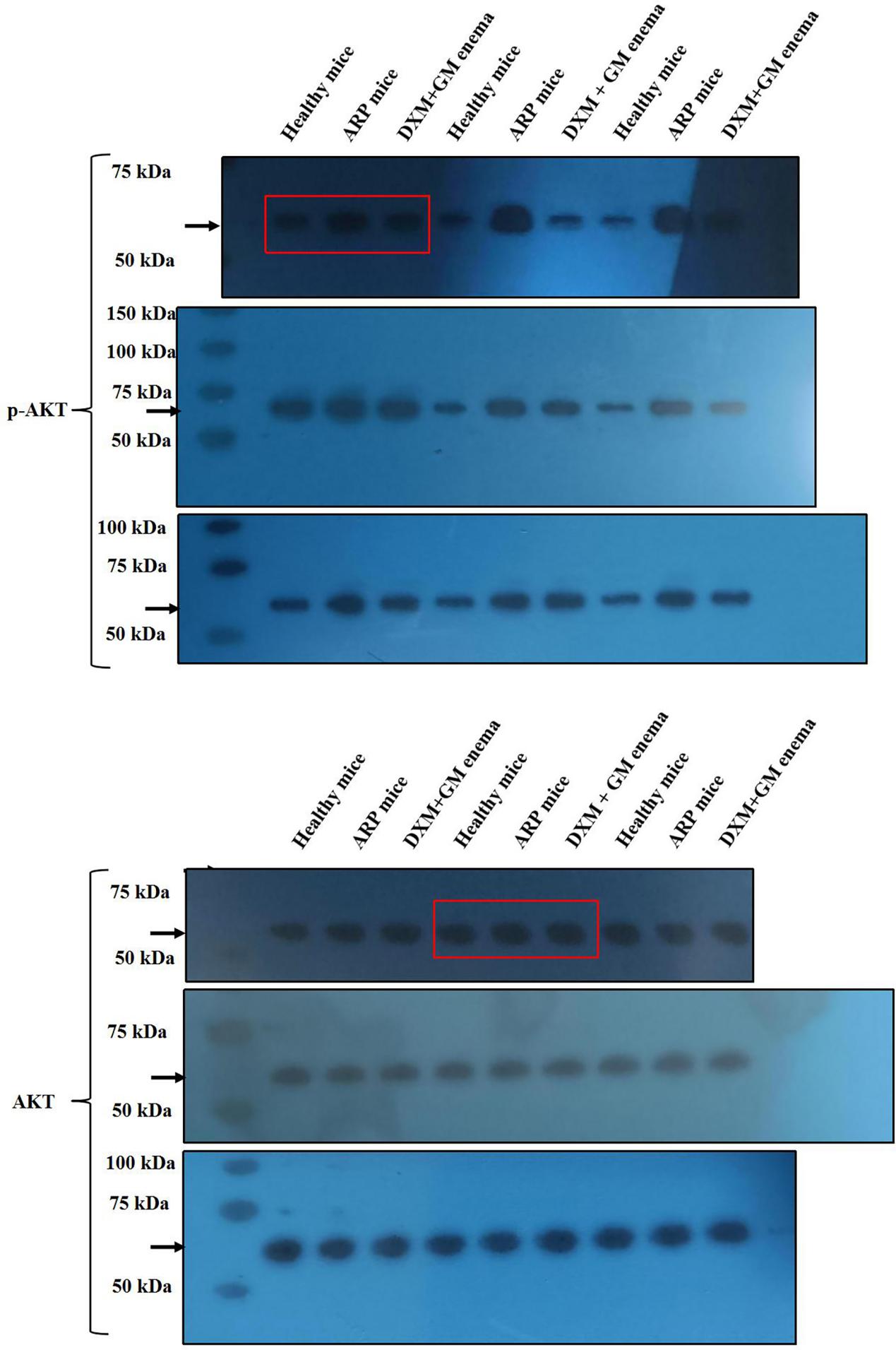

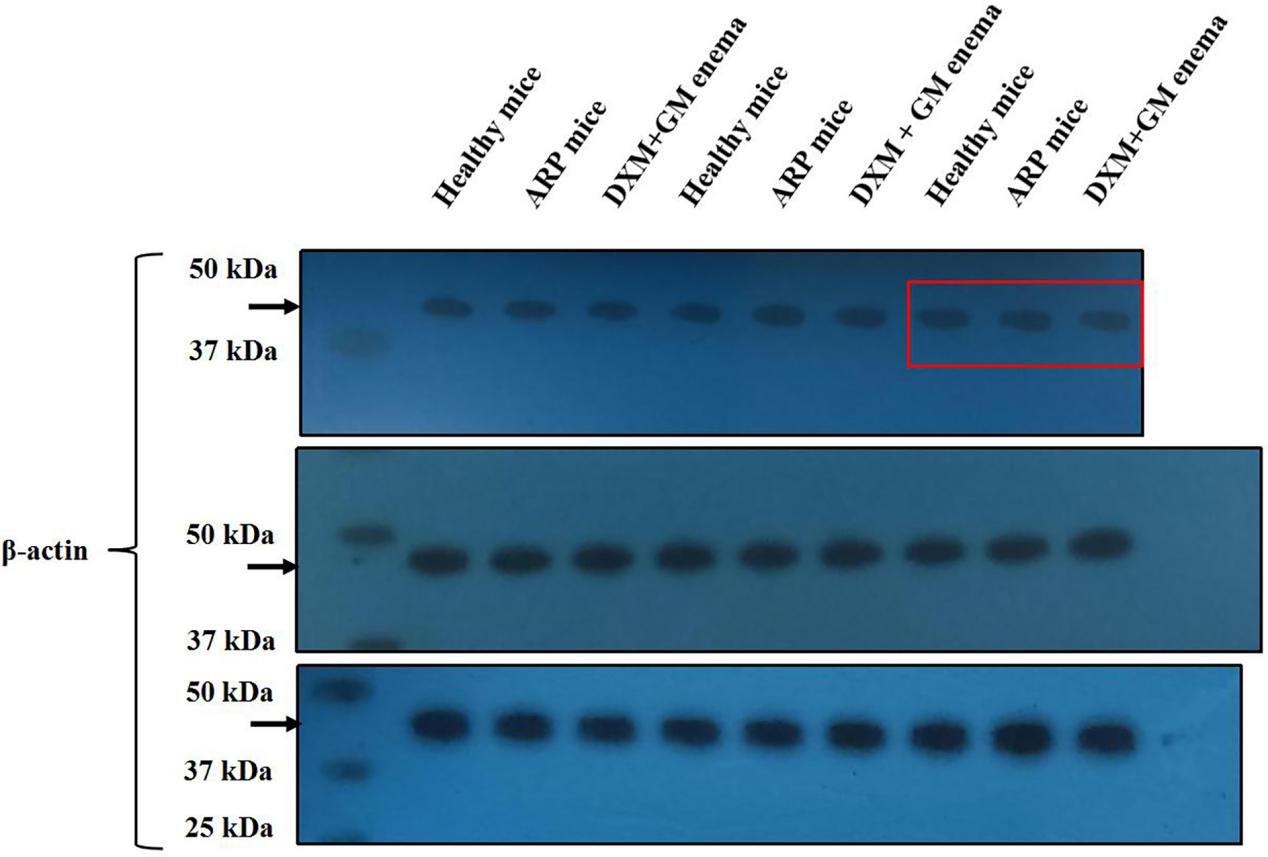


**Figure S4.** ***DXM + GM enema leads to apoptosis in ARP mice*.** The expression of Bcl-2 and Bax protein levels in rectal tissues of ARP mice treated by DXM + GM enema using western blotting. β-actin was used as loading internal control. The original images shown derive from the triplicate experiments. The list as followed as healthy mice, ARP mice and DXM + GM enema. Membranes were cut based on the size marker and hybridized with different kinds of antibodies when needed. The red boxes indicate the cropped areas shown in Figure 9A.


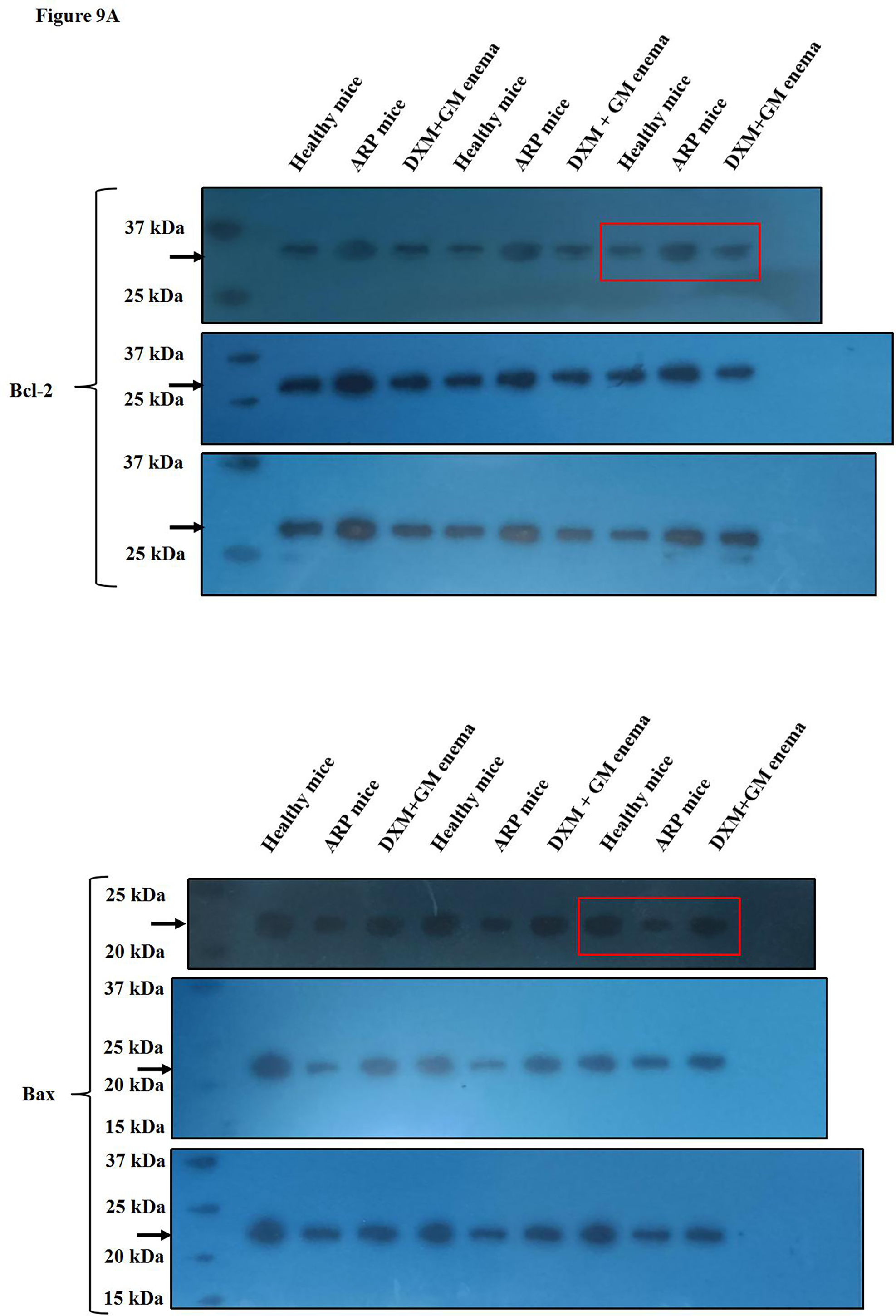


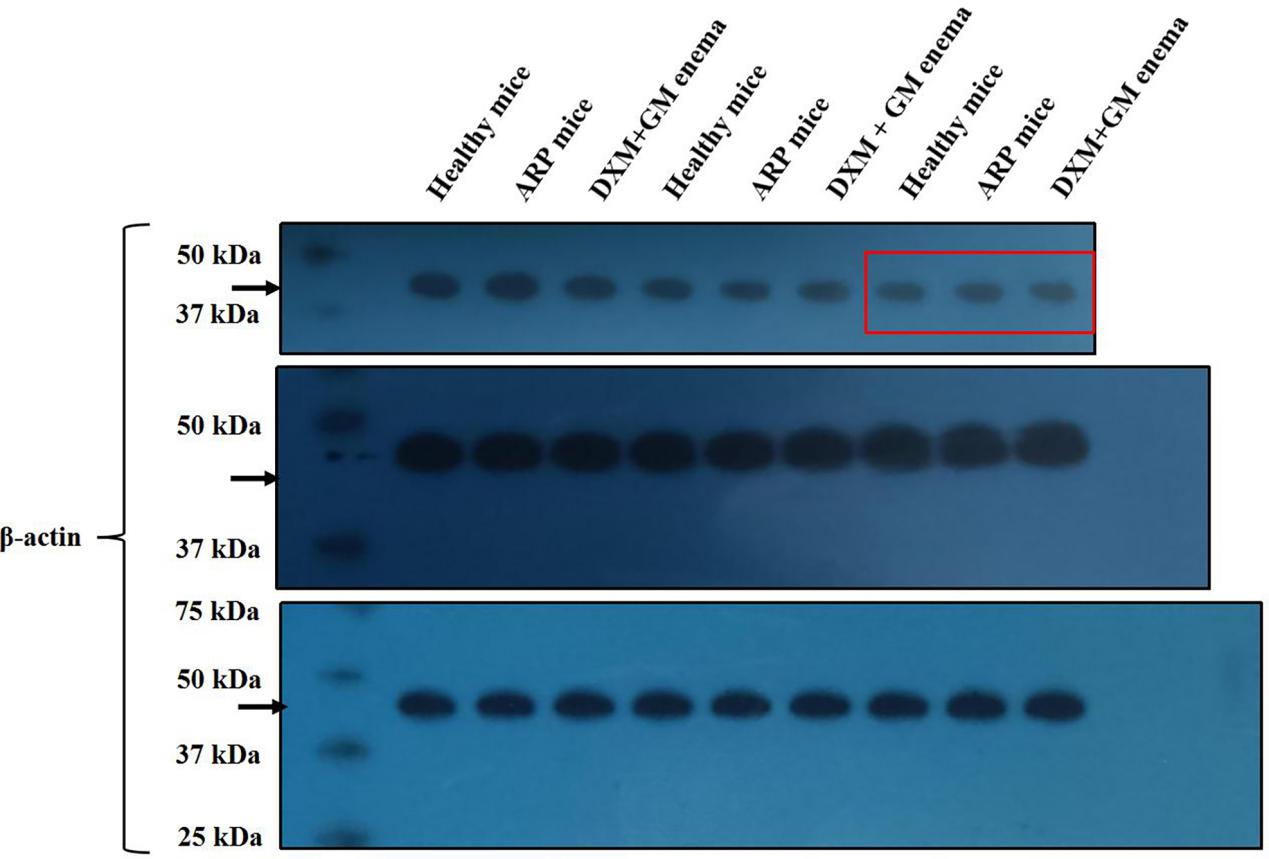

Supplement: Supplementary file 1 — Supplementary Information. [file 41598_2022_17981_MOESM1_ESM.docx]
